# Interactions in multi-pattern Müllerian communities support origins of new patterns, false structures, imperfect resemblance and mimetic sexual dimorphism

**DOI:** 10.1038/s41598-020-68027-w

**Published:** 2020-07-08

**Authors:** Michal Motyka, Matej Bocek, Dominik Kusy, Ladislav Bocak

**Affiliations:** Laboratory of Diversity and Molecular Evolution, Palacky University, 17. Listopadu 50, 771 46 Olomouc, Czech Republic

**Keywords:** Mullerian mimicry, Entomology, Phylogeny, Ecosystem ecology

## Abstract

Mimicry is a hot spot of evolutionary research, but de novo origins of aposematic patterns, the persistence of multiple patterns in Müllerian communities, and the persistence of imperfect mimics still need to be investigated. Local mimetic assemblages can contain up to a hundred of species, their structure can be a result of multiple dispersal events, and the gradual build-up of the communities. Here, we investigate the structure of lowland and mountain mimetic communities of net-winged beetles by sampling the Crocker Range in north-eastern Borneo and neighbouring regions. The local endemics evolved from the Bornean lowland fauna which is highly endemic at the species level. We inferred that metriorrhynchine net-winged beetles evolved in high elevations yellow/black and reticulate aposematic high-contrast signals from a widespread low-contrast brown/black pattern. As the mountain range is ~ 6 million years old, and these patterns do not occur elsewhere, we assume their in situ origins. We demonstrate that a signal with increased internal contrast can evolve de novo in a mimetic community and can persist despite its low frequency. Additionally, a similar aposematic signal evolves from different structures and its similarity is imperfect. The community with multiple patterns sets conditions for the evolution of aposematic sexual dimorphism as demonstrated by the yellow/black male and reticulate female pattern of *Micronychus pardus*. These insights elucidate the complex character of the evolution of mimetic signalling in the dynamically diversifying biota of high tropical mountains.

## Introduction

Mimicry, as an anti-predatory strategy, is one of the textbook examples of improvement through natural selection^1,2^. The model of Müllerian mimicry proposes that the numbers play an important role when a fixed number of unprofitable preys is attacked. Therefore, being unprofitable, aposematic and a member of a large community decreases the probability of an attack for each individual^3^. To share the attack load, protected mimics are selected for such a degree of similarity that potential predators are sufficiently informed about their unprofitability^4^. Due to the frequency-dependent selection, all prey should become sooner or later closely similar, i.e., all should get the best available protection against well-informed predators^5–7^.

The research is often directed to the understanding of interactions of a single predator and a very limited number of prey. In contrast, the real mimetic communities are more complex, and the characteristics of some mimetic systems deviate from model predictions. The Müllerian rings often contain prey displaying several distinct patterns and the mimetic similarity of a high number of species, often only distantly phylogenetically related, can be imperfect^2,4^. As a result, we may assume that the complexity and uncertainty of the signal can increase the probability of laps in predators’ decision-making^8^.

Further often discussed question is the origin of a new pattern. The initial phase of its presence in an ecosystem means initial low numbers of signalling individuals and as a result a higher predation load per individual for early members of a new mimetic pattern^9–11^. Additionally, some aposematic signals might be less effective^12,13^. The low contrast patterns are remembered for a short time and a predator must commonly encounter such prey to preserve the learned avoidance^12,13^. The high internal contrast, uniqueness, and conspicuousness of a potential prey should be easily remembered^14–17^. Broadly, although the purifying selection should favour those who are best protected, i.e., those who share the most common and most effective signal in the community, the real multi-pattern mimetic complexes contain mimics with diverse signals components and imperfect signalling.

Within these complex mimetic Müllerian communities, there is apparently a grey zone between prey which should be highly or poorly protected according to the results of experiments under controlled conditions. The selection in complex mimetic systems can be relaxed for a high number of reasons and, at least temporarily, some poorly adapted mimics can survive. In a decision-making process, the predator can assign prey to more inclusive categories and all brightly coloured prey can be avoided at some level^4,18^, the composition of the predator community can change, some predator can perceive the signal differently^19,20^, and the signal effectiveness may vary depending on conditions and behaviour^18,21^. As a result, we encounter multi-pattern communities in nature and in the contrast with the model-based predictions, aposematic patterns can be conspicuous in various degrees, some mimetics can be perfect and some only vaguely resemble the dominant unprofitable prey. Conspicuousness and perfection are ultimate adaptations of any member of the Müllerian community which tries to maximize protection and minimize costs, but under real conditions, they seem often unattainable.

Here, we study South-East Asian net-winged beetles (Coleoptera: Lycidae) which are common in humid tropical forests^22^. The evolution of mimicry in these elateroid beetles has not been intensively studied, but the whole family is known for aposematic coloration and protection by smelling and bitter compounds^23,24^, usually resemble each other in a given place and are often mimicked by other insects^25–27^. Unlike the traditional model of *Heliconius* butterflies, the communities of these beetles contain more species (up to a hundred species in a limited area compared with a few species of *Heliconius*^21,28^) and the members of the complexes are phylogenetically more distantly related (origin of *Heliconius* co-mimics less than 1 million years ago (mya), the net-winged beetles co-mimics belong to lineages which split up to 100 mya^29,30^). Contrary to *Heliconius*, our knowledge on lycids’ predators, behaviour and genomics is limited and our conclusions are be necessarily weaker than those based on better studied groups. Nevertheless, the characteristics of real multipattern systems should be studied to understand various evolutionary processes in their complexity.

**Figure 1:**
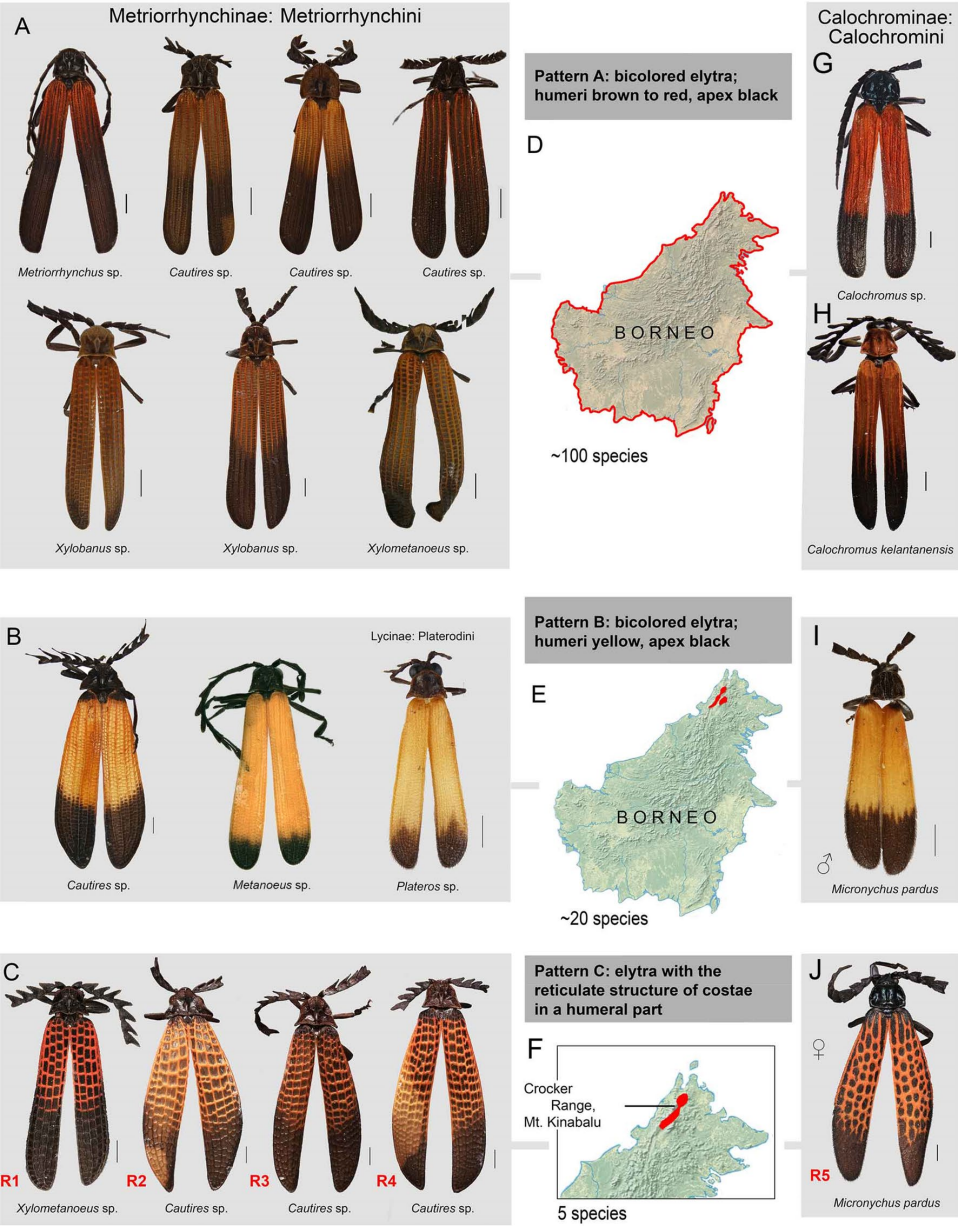
(**A**) the examples of the lowland brown/black and red/black Metriorrhynchini pattern A. (**B**) the examples of the highland yellow/black mimetic pattern B; left *Cautires* sp. (Metriorrhynchini: Cautirina), middle *Metanoeus pendleburyi* Kleine (Metriorrhynchini: Metanoeina), right *Plateros* sp. (Lycinae: Platerodini). (**C**) the examples of the highland reticulate mimetic pattern C; the codes R1–R4 designate the respective species in Fig. 3A. (**D**–**F**) maps of Borneo; from above D, the distribution of the lowland brown/black pattern A; E, yellow/black pattern B; and C, the reticulate pattern C. (**G**–**J**) the examples of the Calochromini co-mimetics, from above G, red/black form of the pattern A, (**H**) brown/black form of the pattern A; (**I**) yellow/black pattern B, and (**J**) reticulate pattern C. The code R5 is used to show the phylogenetic position of *Micronychus pardus* in Fig. 3B. The maps were downloaded from Natural Earth server (https://www.naturalearthdata.com) and edited using Adobe Photoshop CS6 (https://www.adobe.com/products/photoshop.html).

We focus on these beetles in the Mt. Alab and Mt. Kinabalu, two high elevation regions of a tectonically young tropical mountain^31,32^. The local lycid community is very diverse and several dozens of metriorrhynchine net-winged beetles have already been reported in these mountains^22,33^. Most local net-winged beetles display widely distributed aposematic pattern with small elytral cells whose structure cannot be seen from a distance. Therefore, most net-winged beetles display a pattern whose principal component is colour and rather low contrast between brightly and black parts of elytra (Fig. 1A). The internal contrast can be higher if the brightly coloured part of elytra is yellow (known from the Crocker Range and Trus Madi massif; Fig. 1B). Only in the north-eastern Bornean mountains occur some species with the highly conspicuous reticulate structure of elytral costae^34^ (Fig. 1C).

Our aim is to investigate the origin of the high-contrast, presumably easily remembered, signals which we assume as highly effective^13–16,35^. Specifically, we ask how many species display such signals, how closely they are related, when the signals evolved, and where it occurs. Further, we intend to investigate, how extensive are local mimetic communities concerning the number of aposematic patterns, and what proportion represent the species displays unique high-contrast patterns. Although the common name designates Lycidae as 'net-winged beetles', not all of them have a reticulate structure of elytral costae. On the contrary, a large subfamily of lycids, the Calochrominae, does not have any transverse costae and their longitudinal costae are weak, inconspicuous and never modified to produce contrasting patterns. Therefore, we ask what if a net-winged beetle without transverse costae in its elytra has a chance to join the complex of aposematically coloured unprofitable distant relatives that advertise their unprofitability by the presence of conspicuous reticulate costae with large cells. If the beetles who never had transverse costae join the mimetic ring with reticulate costae and the natural selection produces their 'false costae', would these costae be perfect and highly similar to the real ones? We are further interested in the question if the body size plays any role in the signalling. If the male body, unlike the female one, is so small that the reticulate pattern cannot be appropriately shown, do the male nevertheless adopt the reticulate pattern? If not, can the males and females follow different evolutionary pathways in multi-pattern mimetic communities? These questions have several connotations: they can provide evidence for a de novo origin of a new high-contrast aposematic signals, suggest if imperfect mimics get sufficient protection to survive for a prolonged period and if multi-pattern communities can effectively select for sexual dimorphism. We believe that phylogeny, dated trees and the information on mimetic communities can provide plausible answers to our questions. Further, we ask if related species sharing similar body characteristics adopt similar evolutionary pathways in multi-pattern Müllerian communities or if the evolution of mimetic signal is stochastic. The deviations from predicted outcomes are complex and only the structure of real mimetic communities can indicate how the opportunities are exploited by the evolution.

## Results

### Phenotypic classification of Bornean Metriorrhynchini and Calochromini

**Figure 2:**
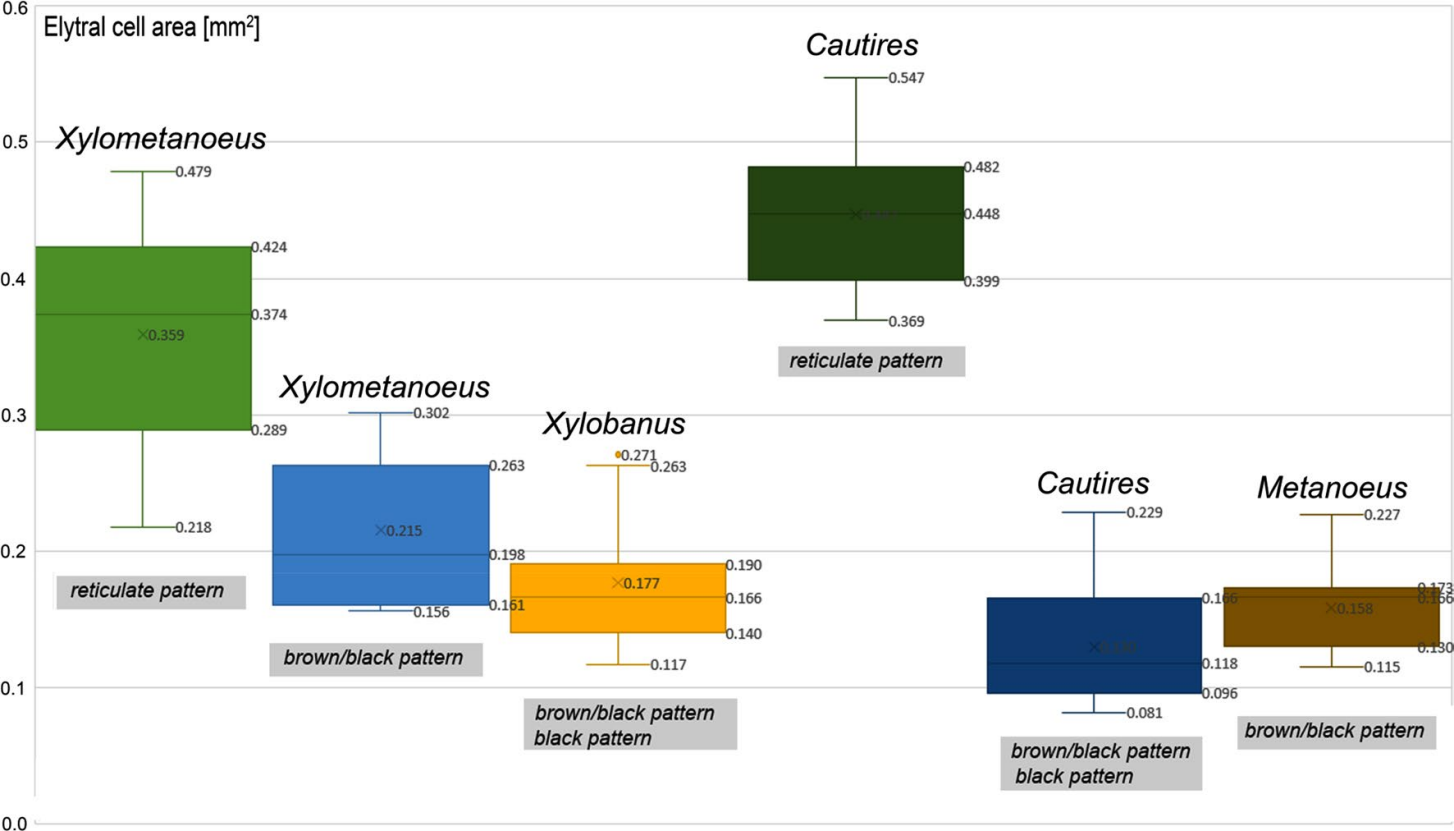
The box plot showing elytral cell area in mm^2^ of various genera displaying brown/black and reticulate patterns. In the box plots, the low boundary indicates the 25th percentile, a line within the box marks the median, and the upper boundary indicates the 75th percentile. The lower whisker represents minimum values and the upper whisker represents maximum values.

**Figure 3:**
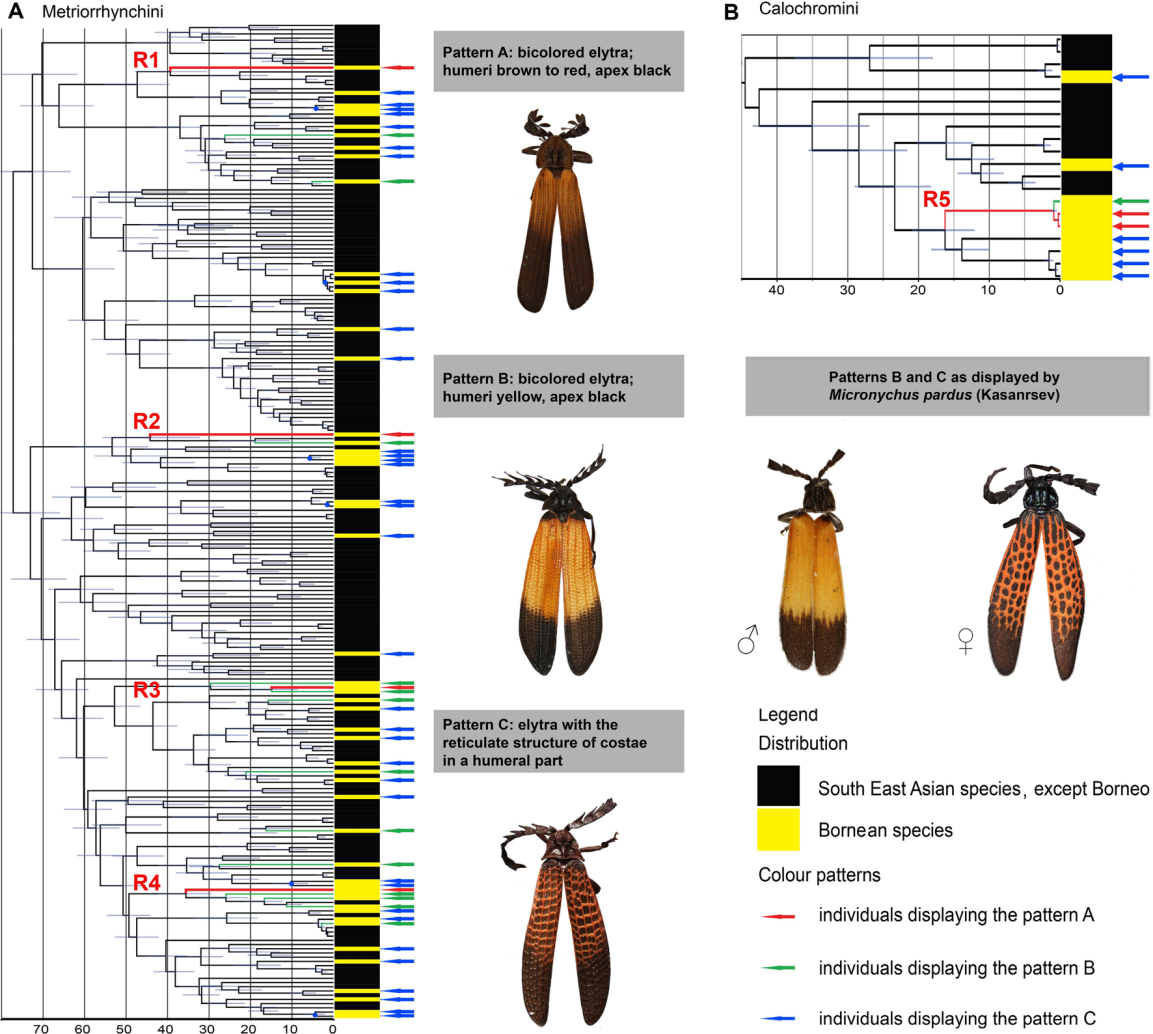
(**A**) The dated phylogeny of the Metriorrhynchini tribe. The red-coloured branches R1–R4 represent the lineages with reticulate pattern shown in the Fig. 1C. (**B**) The dated phylogeny of the Calochromini, the red lineage represents *Micronychus pardus* (R5 in Fig. 1E). The yellow bars at terminals in figs (**A**) and (**B**) designate Bornean species and the arrows at terminals designate aposematic patterns A–C which are shown in the photograph to the left: blue arrow—the brown/black pattern A, green arrow—the yellow/black pattern B; red arrow—the reticulate pattern C.

The colour patterns of Bornean Metriorrhynchini are classified into three categories:

1. *Pattern A* The elytra brown to red in a humeral part of elytra, the apex of elytra dark coloured, the elytral cells minute, elytra with usually > 30 transverse costae in the length of elytron (Fig. 1A). The pattern is considered as a low-contrast type (humeral and apical part of elytra DeltaE 15.7–20.3, i.e., the colours apparently more similar than opposite). The change between humeral and apical colouration can be gradual without the apparent border between bright and dark parts. Some species display a difference in the colour of costae and cell bottoms in the humeral part of the elytron (DeltaE 23.5–28.8).
2. *Pattern B* The humeral part of elytra yellow, the rest of elytra black, with ~ 30 transverse costae in the length of elytron (Fig. 1B). The pattern is considered as a high-contrast type (the colours of the humeral and apical part of elytra more opposite than similar, DeltaE 52.5–69.7). The colouration of the brightly coloured part of elytra can be almost uniform (perceptible differences between the humeral and middle part of elytra DeltaE 3.3–5.4, *Cautires* sp., *Metanoeus* sp., *Plateros* sp.) or the humeral part is darker, slightly red component shifted (e.g., at humeri the colour La*b* CIE76 position 52/33/51 versus the middle of the elytron colour position 64/25/52). The DeltaE colour distance between humeral and middle part of the elytron can reach up to 22.2 and is apparent at a glance.
3. *Pattern C* The humeral part of the elytra with a reticulate structure of four longitudinal and ≤ 20 transverse costae, the apical part dark coloured (Fig. 1C). Species with the elytral reticulate pattern showed more than four-times larger cell volume area in *Cautires*, and twice larger in *Xylometanoeus* compared to related species without the reticulate pattern (Fig. 2). The colouration of the humeral part of costae is bright and the colour varies between a light yellow hue (the La*b* Cie76 colour position 53/19/43) and a reddish bright hue (the La*b* CIE76 colour position 40/27/30). The DeltaE colour distance between costal and cell colouration varies between 25.0 and 40.9. No sexual dimorphism was identified in Metriorrhynchini and similar patterns are identified in unrelated species (Fig. 3A). Some Metriorrhynchini in the region are uniformly black coloured and these are not discussed further.

The colour patterns of Bornean Calochromini are classified into the same three categories with the given difference:

- (1), (2) Patterns A and B: as in Metriorrhynchini (Fig. 1G–I).
- (3) Pattern C: the elytra with a reticulate structure of four longitudinal and 8–10 transverse ‘costae’, superficially similar to the pattern C of Metriorrhynchini. As *Calochromus* does not have true transverse costae, its ‘false costae’ are formed by the uniquely arranged pubescence (Fig. 1J). Most species display the pattern A. The patterns B and C are known in males and females of *Micronychus pardus*^31^. Its female belongs to the pattern C, i.e., resembles the metriorrhynchine reticulate pattern C and the male belongs to the pattern B, i.e., the metriorrhynchine and platerodine yellow/black pattern B (Figs. 1I, J).

The colour interspecific variability in the pattern A is higher in the brightly coloured parts than in dark ones (DeltaE up to 13.5 versus 4.6, respectively). For the pattern B, a higher colour distance was identified between the colour of humeri of individual members of the pattern (DeltaE up to 25.5, average 13.1) than between middle parts of the elytra of respective species (DeltaE up to 11.0, average 6.5).

### Phylogenetic analysis

Using simultaneous consideration of morphology and mtDNA-based relationships we identified 236 species of Metriorrhynchini in the sequenced material, 53 of them from Borneo. Our maximum likelihood analyses recovered a tree with poorly to moderately supported deep splits (Supplementary Figs. S1, S6). Genus-ranks terminal clades were well-supported as well as splits among terminal taxa, which are relevant for further discussion. The species sharing distinct patterns evolved independently in unrelated lineages (Figs. 3A, B, S5): four Metriorrhynchini species with the conspicuous reticulate pattern patterns represent isolated single-species terminals (designated with letters R1–R4 in Figs. 1C, J and 3A, B). Similarly, all thirteen yellow/black coloured species were inferred as single terminals (Fig. 3A).

The dated phylogenetic tree is shown in Fig. 3A, B and Supplementary Fig. S4. The high-contrast yellow/black and reticulate patterns are represented only by terminals and as a result of independent origins in single terminals, we are not able to precisely date them.

The phylogeny of Bornean Calochromini contained 20 terminals representing 15 species, 5 of them from Borneo (Fig. 3B, Supplementary Figs. S2, S3). The dated tree identified the origin of the reticulate pattern only in the females of *Micronychus pardus* which split from the closest relative in the analysis ~ 15 mya (the clade designated as R5; Fig. 3B). The male of this species resembles unrelated species of a yellow/black *Plateros* sp. and the yellow/black Metriorrhynchini which have a slightly larger body (Fig. 1B, I).

### Alpha diversity of the Bornean fauna

The samples represented 150 Oriental metriorrhynchine species. The turnover between major islands and zoogeographically delimited continental areas is high with only 8 species recorded in two neighbouring regions (Supplementary Table S4). The Bornean material contains 53 metriorrhynchine and 5 calochromine species (Supplementary Tables S5, S6, S7) and 4 species were simultaneously collected in Borneo and the Malay Peninsula (two *Metriorrhynchus*, one *Cautires*, and one *Xylobanus*). We identified complete endemism of the mountain biota in the Crocker Range, i.e., Mt. Emas and Mt. Alab (14 species) and the west slope of Mt. Kinabalu (Kundasan and Mesilau, 9 species). Another 22 species were recorded from lower elevations surrounding the mountainous area, mainly from Poring (16 species), and additional 6 species in other localities. The individual localities yielded up to 59 individuals and 16 species (Supplementary Tables S5, S6).

The black and brown/black patterns were mostly recorded in the lowland localities. The reticulate and yellow/black patterns were dominantly recorded in elevations over 1,500 m a. s. l. (Fig. 1C, Supplementary Fig. S6, Supplementary Tables S5, S6). The reticulate co-mimics (pattern A) represented ~ 30% of individuals in mountain communities and they were represented by four species of Metriorrhynchini. The yellow/black pattern B occurred in the same localities, the species displaying this pattern represented ~ 50% of individuals and 13 species (Supplementary Table S5). Other individuals belong to the brown/black pattern A and they become less common with the increasing elevation. All Metriorrhynchini showed a limited flying activity and their adults commonly aggregated in some spatially limited areas where a higher abundance of net-winged beetles was recorded. The distribution of mimetic patterns recovered by the study of the material used for the molecular study was confirmed by the thorough investigation in major European collections and the collection of the Sabah National Parks in Kundasan.

Our sampling contains 15 Calochromini species, 5 of them from Borneo (Fig. 3B). The reticulate pattern C was identified in one species *M. pardus* (females only). Its male displays the yellow/black pattern B. Only three females and one male of *M. pardus* have been collected until now^30,31^.

## Discussion

Most studies on Müllerian mimicry have focused on behaviour, predators' choice, and modelling, usually under controlled conditions. We suggest that the phylogeny and structure of real Müllerian communities can test experimental results and elucidate the origins of numerous patterns, their long-term coexistence, imperfect similarity, and mimetic polymorphism^7,8,11,35^. A similar approach has been used for studies on velvet ants^36^ and, here, we focus on the evolution of aposematic patterns in net-winged beetles. We base our study on the structure of Müllerian complexes and the phylogenetic analysis of the Bornean fauna, including its relationships with other Oriental Lycidae (Fig. 3A, B).

The Bornean fauna is highly endemic, and our samples contain 53 Metriorrhynchini and 5 Calochromini species, only 4 of them known from neighbouring regions. Molecular data, as well as morphological divergence, indicate that the closest relatives are present in the Malay Peninsula and Sumatra and that the divergence between species justifies species rank for most species pairs endemic to respective areas as has been shown in the earlier detailed study of the net-winged beetle faunas of the Malay Peninsula and Sumatra^37,38^ and similarly reported in vertebrates^39^. Our sampling is undoubtedly incomplete as we analysed only 53 Bornean metriorrhynchines species compared to 69 formally described. Nevertheless, our results convincingly show that high numbers of species are involved in each Müllerian complex in each locality and that they belong to various deeply rooted lineages. Due to distant relationships of Müllerian mimics in Borneo, we suppose that their similarity is a result of natural selection, not relationships (Fig. 3A, B).

We observed individuals belonging to multiple patterns in close contact in the mountain forests of the Crocker Range. Most individuals were sampled in aggregations on a limited number of shrubs and low-stratum trees in the forest. Additionally, the mountain forests in the Mt. Crocker Range represent a geographically very limited area (Fig. 1F). The area defined by 1,500 and 2,500 m contour lines covers ~ 140 km^2^ in Mt. Kinabalu and ~ 60 km^2^ in the Mt. Alab – Mt. Emas area. The mountains species are endemic to such a small range as the mountains of north-eastern Borneo are highly isolated and net-winged beetles poorly dispersing^37^. The occurrence in aggregations and a very small, ecologically uniform range indicate that the observed patterns coexist in contact and are not isolated in non-overlapping microhabitats as has been observed in some butterfly mimetic systems^40,41^.

Broadly defined, three mimetic patterns are identified in Borneo, including the mountain areas of the Crocker Range. Both tribes, Metriorrhynchini and Calochromini contained species sharing all three patterns. The brown to reddish/black pattern A (Fig. 1A, Supplementary Fig. S6) dominates in the Bornean lowlands and lower mountain elevations up to ~ 1,400 m (Fig. 1D). Although similarly coloured, as the component of the signal can be considered also the similar body size and shape which provide at least limited signalling of unprofitability when an individual is sitting on the bottom side of a leaf and is observed against clear sky^21,42,43^. The extent and shade of the bright part of elytra is intra- and extra-specifically variable, but most individuals have one to two-thirds of elytra brightly coloured and these individuals can be categorized as a single pattern (the colour differences are perceptible at a glance; average DeltaE 9.87). Further, we identified the presence of two unique mimetic patterns in the elevations over ~ 1,500 m (Fig. 1E, F, Supplementary Fig. S6). The mountainous yellow/black pattern B is quite close to the widespread lowland red/black pattern and some individuals have shades of red in some parts of the yellow coloured humeri (Fig. 1B and see results for La*b* CIE76 colour positions). We assume that such a pattern could evolve from the lowland forms by a simple modification of coloration. Although some intra-pattern variability is perceptible (Fig. 1), these patterns can be easily assigned to categories and serve as a warning signal for potential predators. Their signalling role is supported by the presence of co-mimics from other beetle families and insect orders^43,44^.

**Figure 4:**
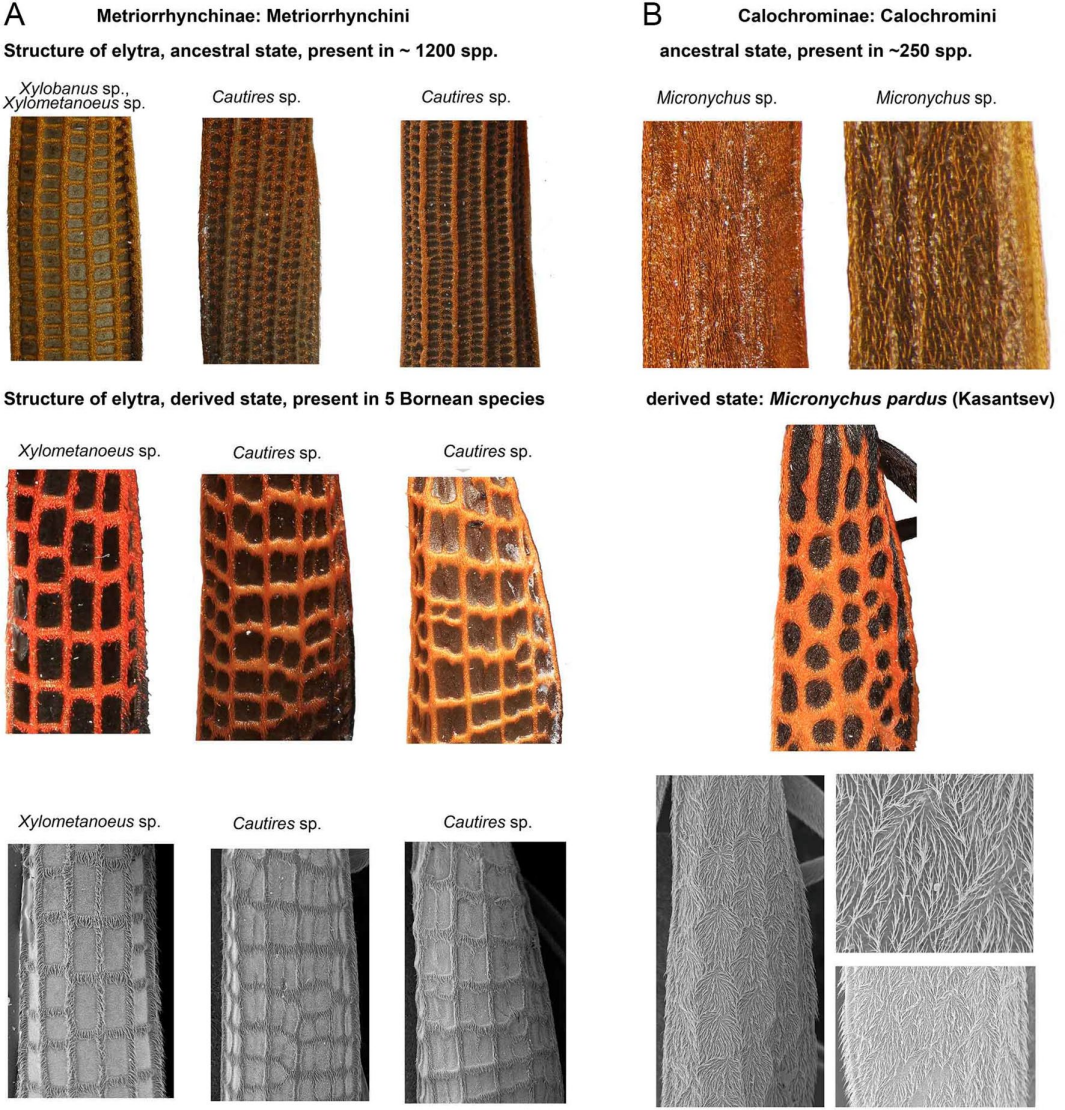
(**A**) The examples of the metriorrhynchine and calochromine elytral structures using light macrophotography and SEM photographs of the homological elytral parts. The genera for columns are given above, the second and third row show always the homologous parts of a single specimen. The first column shows the comparison of species with different number of transverse costae with the size of cell increased in derived forms, the second and third column show examples of the lost and/or vestigial secondary costae and increased size of cells as a result. (**B**) ditto of Calochromini. Above, the examples of the typical structure of costae in Calochromini. Middle and bottom, the examples of the female *Micronychus pardus* elytral structures using light macrophotography and SEM photographs (the ‘false’ reticulate costae resembling the pattern C).

The reticulate pattern C involves, besides a colour component, also the structure of elytral costae and it is the unique, easily distinguishable distinct signal of unpalatability (Fig. 1C). All Metriorrhynchini have either four or nine longitudinal, and numerous transverse costae connecting them (Figs. 1A–C, 4A). The relationships of the species displaying the reticulate pattern show that such a pattern has to be derived from the ancestral small-cell type. *Xylometanoeus* and *Xylobanus* have four costae even if they are uniformly black or brown/black, but either the number of transverse costae is high and as a result, the cells are small and cannot be distinguished from a distance (Fig. 1A) or even when cells are slightly larger than usual, the colouration of cells does not differ from those of costae and they cannot be distinguished from species with nine costae at the first sight. The *Xylometanoeus* species with the reticulate aposematic pattern has a much lower number of transverse costae, the costae are covered by red pubescence, and the large cells with black bottoms are very apparent (Figs. 1C, 2). The reticulate pattern is displayed also by some *Cautires* species. Almost all *Cautires* have nine longitudinal costae, but when these beetles display the reticulate pattern, their secondary costae are reduced. Such adaptation produces relatively large cells, nevertheless, the cells are smaller than those of *Xylometanoeus* (Figs. 1C, 2, 4A). Our phylogenies show that the reticulate pattern is a derived trait. We may suppose a gradual evolution of increasingly conspicuous reticulate cells as transitional forms are present in the pattern A (Delta E colour distance costa/cell 23–30 in some representatives of the pattern A and 25–41 in the pattern C). The signal is primarily based on the reticulation. Even if costae and bottom differ in colouration, more apparent is their different size (compare Fig. 1A, C).

The dated tree identified the splits between individual species displaying the focal reticulate and yellow/black patterns and their closest relatives a long time before the uplift of the Crocker Range (15–45 mya in Metriorrhynchini and ~ 15 mya in Calochromini). As each species represents a single lineage and no split between species sharing the reticulate pattern was identified, we are not able to date reliably the origin of the pattern. Better data could be potentially obtained with denser sampling, which is currently unavailable. Therefore, we must rely on indirect evidence to date the origin of patterns. The Crocker Range is a tectonically young mountain area and was uplifted in the last ~ 7.5 million. The net-winged beetles are common and diverse in high-mountain areas which obtain high rainfall, i.e., in the montane forests established in the area at the earliest 6 mya^22,31,32^.

### The origins of high-contrast patterns in a mimetic community

The yellow/black and reticulate patterns B and C show a higher Delta E colour distance, i.e., internal contrast, than the widespread brown/black pattern A (Figs. 1B, C, 4A, B). The high-contrast patterns were observed in several species of Metriorrhynchini and a single species of Calochromini and Platerodini; other net-winged beetles do not display them. The reticulate pattern C was identified in 5 species, 4 metriorrhynchines and 1 calochromine, the yellow/black pattern B in 13 metriorrhynchine species, 1 *Plateros*, and 1 *Micronychus* (Fig. 1B, I). The phylogenetic analysis shows that all of them represent terminal lineages (Fig. 3A, B). As the reticulate and yellow/black patterns do not occur outside the north-eastern Bornean mountains, we must suppose their de novo sympatric origin within the earlier local mimetic communities which display widely distributed pattern A in Borneo as a whole and in most of south-east Asia.

The reticulate structure of elytral costae is apparently derived from patterns displayed by their closest relatives^35,36^. In the principle, the distinctiveness of the reticulate pattern is based on the lower number of costae (four instead of nine longitudinal costae) and a lower number of transverse costae in the length of the elytron (8–15 versus > 30 transverse costae in patterns A and C, respectively). As a result, the elytral cells are much larger, 0.29–0.48 mm^2^ compared to 0.10–0.26 mm^2^ in non-reticulate elytra (Fig. 2). Additionally, the dense pubescence of costae is bright coloured and the cell bottoms are black (Delta E 25.0–41.0 between costae and cells in the pattern C; DeltaE up to 28.0 in pattern A; Fig. 3C, J).

Similarly, the yellow and black coloured parts of elytra in the pattern B display a higher internal contrast than reddish brown/black pattern A (Delta E 52.5–69.7 in the pattern B and 15.7–22.2 in the pattern A, respectively). Therefore, as perceived by a human eye, the pattern B is more conspicuous than the dominant and widespread reddish brown/black pattern A of relative species (Figs. 1, 3, 4). Its distinctness is based on a higher internal contrast between bright and black parts due to the bright yellow pubescence and light colouration of costae and cells in the basal part of elytron (Figs. 1, 4). High contrast and structural uniqueness are principal components of the signal and increase its effectiveness^13–16,35^. We can expect variable perception under different levels of illumination and variable external contrast on different backgrounds (old dark coloured leaves versus young thin light green leaves). Although, the perception might vary under different conditions, the colour differences are perceptible and can be quantified.

In the contrast with expectations based on frequency dependent purifying selection^6^, we observe a relatively low numbers of individuals displaying the reticulate pattern C and most individuals belong to a single species of *Xylometanoeus* (Fig. 1C) and other four species were collected in few specimens (Table S1). Therefore, we assume that species displaying the reticulate pattern C do not have the full protection of a high number of individuals. Their relatives with whose they undoubtedly shared the widespread and very common reddish/brown pattern a few million years ago absolutely dominate in the lower elevations. The theory-based expectation is that the species displaying a less common pattern suffer a higher attack rate if they encounter uninformed predators. The disadvantage of the rareness must have been even stronger in the early phase of the evolution of these patterns when the first more conspicuous distinct forms evolved, and a low number of individuals displayed the new pattern. We have to consider some factors potentially supporting survival of such conspicuously coloured individuals and compensating the disadvantage of the low number of individuals. The only potential benefit, which the members of a new mimetic ring attained due to their new high-contrast aposematic signal, is possible predators' rapid ability to associate unprofitability with their unique reticulate pattern and the ability to retain such association for a long time^6,14,45–48^. Therefore, as a working hypothesis, we assign the apparent success of such a new pattern to its high internal contrast which putatively facilitates avoidance learning and offsets the disadvantage of low numbers.

The survival of conspicuous prey unknown to local predators has been a conundrum of the mimicry theory^3^. The here described process suggests that it is possible to abandon an extensive mimetic ring and to sympatrically establish a new distinct pattern despite the predicted higher selection load of supposed higher attack rate per capita from local predators. Therefore, we suggest that new aposematic patterns can be potentially established in original Mullerian communities if the new aposematic signals are sufficiently strong, *e.g*., easily learned by local predators. As a consequence, we must additionally suppose that mimetic communities continually expand. The number of species in the Müllerian ring rises due to advergence of additional species to local patterns and by the sympatric origins of new patterns.

The intraspecific polymorphism is a possible adaptation in multi-pattern communities as has been shown recently in another metriorrhynchine genus *Eniclases*^21^, but the recovered phylogeny indicates that the acquisition of a new pattern was regularly accompanied with the speciation. In such a way, the alpha-diversity diversity was putatively generated in situ in the Crocker Range^32^. Probably due to a limited dispersal capacity, the conspicuous endemic patterns of net-winged beetles are restricted to very small ranges as we observe in the Crocker Range and earlier in Sumatran and Malayan mountains^37^. The occurrence of the new reticulate pattern is limited in the Crocker Range to an area between 1,500 m and the upper limit of the montane forest, i.e., to ~ 200 km^2^ (Fig. 1F). The small range and a low capacity to colonize geographically distant ecosystems are, in the long term, a disadvantage which may lead to extinction if original habitats are lost.

### How to overcome constraints in multi-pattern communities

Our previous discussion focused on metriorrhynchine net-winged beetles. One of the species displaying the reticulate pattern, *Micronychus pardus*, belongs to the Calochromini (Calochrominae, a diverse tribe with a cosmopolitan distribution^49^), a distantly related subfamily whose members do not have any transverse costae (Fig. 4B). The absence of a structure that is crucial for the adoption of the local reticulate aposematic signal is undoubtedly a serious disadvantage if the adoption of such a signal would be profitable. Nevertheless, *M. pardus* evolved a unique reticulate structure of setae on their elytra which resembles the real elytral costae (Fig. 4B).

How did *Micronychus* overcome the absence of transverse costae? The ancestral morphology does not indicate the gradual accumulation of cost-free mutations over the range of imperfect signals^6^. As the transitional origin of small cells and their subsequent modifications would be complicated, we assume the direct evolution of a few transverse "costae" to adverge to the reticulate pattern. We intentionally designated the structure as "costae", because, in fact, the perception of costae is merely an optic illusion caused by the different colouration and direction of dense setae on their elytra (Figs. 1J, 4B). We suppose that if a net-winged beetle which does not have any transverse costae adopts such a new reticulate aposematic pattern, the whole process must be rapid and based on the advergence to the already present reticulate signal. The transitional stage would be neither conspicuous nor known to local predators and all predictions suggest that it should be quickly wiped out. The resulting pattern is undoubtedly similar to those of the local Metriorrhynchini. Nevertheless, the similarity is limited by the ancestral morphology and at least to the learned field entomologist serving as a model predator this pattern does not seem perfect. Despite these limits, we must consider calochromine 'false costae' as a stunning example of the power of evolution when structure which should be modified is absent (Figs. 4B). We cannot tell anything about the mechanism leading to such a modification, but the plasticity of setal colours must be high as shown recently by Tiana et al.^50^.

The situation of unpalatable *Micronychus pardus* among other unpalatable species that use their reticulate costae as an aposematic signal seems complicated enough, but if something can become more complicated, it undoubtedly becomes. The net-winged beetles, similarly to most beetles, have quite large-bodied females and small-bodied males. We found that only females of *M. pardus* adopted the reticulate pattern C. The conspecific male (a single individual available) is small-bodied (male 8.1 mm versus female 9.8–12.8 mm) and surprisingly, it does not resemble its conspecific female. The male resembles yellow/black coloured metriorrhynchines^34^, the pattern B (Fig. 1I) and we suppose that the body size plays an important role in the signalling as shown earlier in *Dilophotes* net-winged beetles^44^.

The adaptations of sexually dimorphic *M. pardus* are a stunning example of a very complex evolutionary process when males and females follow different evolutionary pathways in multi-pattern mimetic communities. Both sexes adopt unique patterns within Calochromini, and the females overcome the absence of an important morphological structure. The multi-pattern communities potentially set a very selective environment that exposes its members to various challenges. Some of them, like four metriorrhynchines and one calochromine species established in north-eastern Bornean mountains a new mimetic ring of with reticulate patterns and 13 species of metriorrhynchines, one calochromine and one platerodine the yellow/black pattern. In contrast with them, many closely related species without reticulate pattern co-occur in the area and many in close contact within the same mountain ecosystems. The number of shifts to the new highly conspicuous reticulate pattern is quite low. Although not conclusively, at least fairly convincingly, we may suggest that a good part of mimicry evolution in Bornean net-winged beetles is stochastic and depends on the availability of a mutation in the right place and time. Therefore, considerable time lags can be expected if a species should adopt a considerably different aposematic signal^4^. The delays then contribute to the coexistence of several patterns in a single community of unpalatable beetles.

## Conclusion

Net-winged beetles have never been among popular model organisms for the studies on mimicry and compared to butterflies, we do not know the full spectrum of their predators and genetic mechanisms of aposematic signalling^23,24,51–53^. Conversely, they belong to the unpalatable organisms which form very extensive and complex mimetic communities containing up to a hundred species and ten aposematic patterns in some localities^21^. Our findings in Borneo show that multi-species communities and the high diversity of aposematic patterns are a rule not an exception in net-winged beetles. The multi-pattern mimetic systems expose its members to a complex selective pressure that we are not able to study in experiments or describe by simple models. Here we prove the sympatric origin of two aposematic patterns with high internal contrast within a mimetic community formed by dozens of species with low colour contrast. Additionally, we recover the origin of sexual dimorphism as an adaptation to the multi-pattern environment. We show that the evolution of new patterns is partly stochastic, and only some members of a mimetic ring adopt them despite their supposed co-existence for at least several hundred thousand years. We consider the potential reason for such diversity of aposematic patterns the high number of species, their distant relationships which makes it difficult for some species to copy the dominant pattern, and the expected dynamic structure of communities in the space and time. Additionally, we show that constraints are not absolute, and some species can evolve false structures which are quite closely similar to a mimetic model. But simultaneously we show, that the origin of false structures is unique, the structure cannot closely resemble the model to the intrinsic limitations and that other closely related species despite being exposed to the similar selective pressure, do not follow the same evolutionary pathway. Stochasticity is well-known in evolution^54,55^, but it has been rarely considered as an important factor in the studies on mimicry. The diversity of evolutionary pathways of individual species can be one of the major reasons for the observed deviation of Müllerian mimics from predictions provided by the traditional model.

## Methods

### Material

The material consists of 484 individuals of Metriorrhynchini and 23 individuals used as outgroups (all Metriorrhynchinae) and 51 individuals of Calochromini with 21 outgroups (all Calochrominae). In total, 407 individuals were sampled in South East Asia and among them 178 individuals from six localities in the Crocker Range in Borneo from the 300 to 2000 m above sea level (a. s. l., Fig. 1; Supplementary Table Sl) and further five localities in Bornean lowlands. We sequenced all available Metriorrhynchini from north-eastern Borneo and all Calochromini specimens. All sequenced specimens were fixed in 96% ethanol in the field. Most specimens were collected by the senior author and collaborators individually or by sweeping in areas with a quite high abundance of Lycidae. Additionally, the large collections of Lycidae in the Museum of Natural History, London, Museum and Institute of Zoology, PAN, Warszawa and National Natural History Museum, Paris and Sabah National Park museum, Kundasan were studied to confirm the presence and distribution of mimetic patterns in South East Asia. Altogether, some 4,000 specimens were studied from South East Asia and about 800 specimens from North Eastern Borneo.

### Laboratory procedures

Total DNA was extracted using the phenol–chloroform extraction and Wizard SV96 Purification System (Promega Inc.). Extraction yields were measured using a NanoDrop-1000 Spectrophotometer. The fragments *rrnL* + *tRNA-Leu* + *nad1* (~ 831 bp), *cox1* (801 bp), and *nad5* + adjacent tRNAs (~ 1,359 bp) were amplified. The primers and settings of PCR reactions follow earlier publications^29,56^ and are listed in Table S2. The PCR products were purified using GeneClean (MP Biomedicals, Solon, OH) or PCRμ96TM Plates (Millipore Inc.) and sequenced by an ABI 3130 automated sequencer using the BigDye® Terminator Cycle Sequencing Kit 1.1. Sequences have been deposited in MendeleyData (DOI: 10.17632/wnzyzk3b4x.2). Some earlier published sequenced were included in the analysis (Tables S1, S7).

### Mitochondrial DNA data sampling and phylogenetic analyses

The Metriorrhynchini dataset contained sequences of *rrnL, cox1,* and *nad5* mtDNA fragments 292, 422, and 426 sequences, respectively. The Calochromini dataset contained the same fragments with 38, 70, and 70 sequences. The datasets of Metriorrhynchini and Calochromini sequences were separately assembled and analysed, as these lineages are distantly related^57^ and the length variability of *rrnL* and *tRNA* sequences would complicate alignment. In both analyses, all fragments were aligned separately using MAFFT v. 7.017^58^ in Geneious v. 7.1.9 (https://www.geneious.com) and the concatenated dataset was analysed to infer a phylogenetic tree. We used IQ-TREE v. 1.6.6^59^ to estimate mtDNA phylogeny with the ultrafast bootstrap support (UFboot) set to 5,000 iterations. The best models for each fragment were selected using ModelFinder^60,61^ (Table S3) implemented in IQ-TREE. The complete dataset was pruned to 259 species-level operational taxonomic units (OTU) using a 2.5% uncorrected pairwise distance threshold. The vouchers of closely related terminals were studied to validate the delimitation of OTUs by the morphological distinctness. For simplicity, these units are designated as "species", hereafter (Supplementary Figs. S1–S3). The pruned dataset was reanalysed as above.

### Estimation of divergence times

The dataset pruned to one representative per species was analysed to recover a dated tree using a Bayesian approach implemented in BEAST v. 1.8.2^62^, and the analysis was set to HKY + I + G proposed as the second-best model after the analyses using the GTR + I + G model did not converge. Further, we set Relaxed Clock: Uncorrelated Lognormal and Birth–Death Speciation Process. We produced 5 × 10^7^ generations with sampling every 2,500 generations. Only *rrnL*, *cox1,* and *nad5* genes were analysed and the genes and codon positions were partitioned (Table S3). Each partition was provided with its own parameters. Because the fossil record is absent and relevant splits are younger than 10^7^ years ago (mya), we used the information on the mtDNA mutation rate in beetles to calibrate our topology. We used a mean rate of 0.0115 substitutions per site per million years per lineage, subs/s/my/l) for *cox1*, 0.0177 subs/s/my/l for *nad5*, and 0.0054 subs/s/my/l for *rrnL*^63^. The best topology recovered from the ML analysis of the reduced dataset (Supplementary Fig. S6) was fixed by the guiding tree and switching off tree operators during analysis. Convergence was assessed in Tracer v. 1.7.1^64^ and the first 1.25 × 10^7^ generations were set as burn-in. Datasets for Metriorrhynchini and Calochromini were separately analysed.

### Morphological study

The morphological traits were observed using an Olympus SZX-16 binocular microscope. We evaluated morphological traits commonly used in lycid taxonomy, i.e., the relative size of yeas, the shape of antennae, male genitalia, and coloration to exclude the possibility that close OTUs represent distant geographic isolates or introgressed mitogenomes. Samples were sorted to putative species using colour patterns, and morphological traits and several representatives of each morphospecies were sequenced from localities outside the Crocker Range. The coloration of the pronotum and elytra were recorded, and patterns were grouped into three discrete categories (Fig. 1, Supplementary Figs S5, S6). The photographs for illustrations were taken either using a Canon EOS 700D APS sensor camera attached to the microscope or a Canon EOS 5D full-frame camera and Canon EF MP-65 and EF 100 mm macro lenses. The LED illumination set was used, the white colour was custom balanced, and the camera default colour profile was used for colour management. Only brightness was finely modified while the photographs were processed. For scanning electron microscopy (SEM), all samples were air-dried overnight at 60 °C and mounted onto metal stubs using a double-sided conductive tape. Dried specimens were coated with gold and examined at a low vacuum with an SEM Zeiss LEO 1430VP at 5 kV at the SEM unit of the Zoological State Collection in Munich.

### Evaluation of colour and patterns

The colouration of Lycidae in such large area as South East Asia is very probably perceived by multiple predators with different spectral sensitivities. The potential predators include arthropods (various spiders, mantids, reduviids, etc.), small reptiles such as lizards, and various species of birds^23^ (personal observation). These predators encounter net-winged beetles under very variable conditions defined by the types of ecosystems, light conditions, and prey behaviour. Net-winged beetles can be preyed when sitting on the upper side of a leaf, the bottom side of thin or thick leaves (therefore sometimes only a silhouette can be observed against the clear sky) or they can be preyed when flying. All situations are affected by light intensity, from very poor conditions under the dense forest canopy and mid-day full light conditions in an open space.

Without any exact data on predator spectrum in the given locality and season and also the time spent by aposematically coloured individuals under various conditions, we had to base the initial definition of three colour patterns on the mammal colour vision system, i.e., the personal experience obtained by authors and collaborators during multiple field expeditions to South East Asia. In total, twelve persons took part in the field research from 2001 to 2016, each researcher collecting at minimum for one months in a specific area. Altogether five person/month were spent in the Crocker Range and Mt. Kinabalu and additional 26 person/month in South East Asia, except north-eastern Borneo. The senior author took part in all expeditions of the research group.

To validate the human field perception of aposematic patterns, we took calibrated photographs of five representatives of the brown/black and yellow/black patterns A and B, and three individuals displaying the reticulate pattern C, all from the Kinabalu massif. The coloration of net-winged beetle specimens slowly changes in collections due to exposure to light and degradation of pigments. Therefore, we were not able to use older collection specimens and had to rely on the sequenced samples recently collected for molecular analyses. Specimens were illuminated with three Solux lightbulbs (Tailored Lighting Inc., Rochester, NY) with colour temperature 4,700 K and photographed with Canon EOS M6 camera equipped with Canon zoom lens 18–150 mm f/3,5–6,3 IS STM (zoom lens was used because of different sizes of beetle specimen and colour calibration target). Images were converted from raw to 48-bit tiff format with dcraw program (dcraw.exe -4 -T -w -v IMG_*.CR2; https://osdn.net/projects/sfnet_dcrawnet/downloads/dcraw.exe/), calibrated with IT8.7 calibration target (R110112, www.coloraid.de) using CoCa program with implemented Argyll Colour Management System (www.argyllcms.com) and finally converted to 24-bit sRGB IEC61966-2.1 colour space using perceptual rendering intent. The colour space characteristics of the bright and dark coloured elytral areas were quantified in the La*b* absolute colour space (International Commission on Illumination, CIE76). The colour samples were taken ten times from the area 31 × 31 pixels in various points of both elytra in Photoshop 13.0. The average colour values were computed and the colour distance DeltaE La*b* CIE76 was counted as the Euclidian distance between colour positions in the 3D La*b* CIE76 space. If the costae and bottom of elytral cells were differently coloured, then additionally the costal and cell colours were sampled from the area of 5 × 5 pixels in ten places of both elytra. The colour positions and distances were counted as described above. The colour distances DeltaE were counted also for homologous parts of elytra of individual species and an average for each pattern and elytral area. The pattern C is characterized by the slightly variable colouration of costae (Fig. 1C) and the potential salient component of the warning signal are the large black coloured cells and brightly coloured costae in the humeral part of elytra. Therefore, the pattern C is characterized besides the colour also by the average area of elytral cells (mm^2^) in the high-contrast area of elytra.

## Supplementary information

Supplementary information

## References

[1] Müller F (1879). *Ituna* and *Thyridia*: a remarkable case of mimicry in butterflies. Proc. Entomol. Soc. Lond..

[2] Mallet J, Joron M (1999). Evolution of diversity in warning color and mimicry: polymorphisms, shifting balance, and speciation. Ann. Rev. Ecol. Evol. Syst..

[3] Sherratt TN (2008). The evolution of Müllerian mimicry. Naturwissenschaften.

[4] Edmunds M (2000). Why are there good and poor mimics?. Biol. J. Lin. Soc..

[5] Mallet L, Barton NH (1989). Strong natural selection in a warning colour hybrid zone. Evolution.

[6] Lindström L, Alatalo RV, Mappes J, Riipi M, Vertainen L (1999). Can aposematic signals evolve by gradual change?. Nature.

[7] Ezray BD, Wham DC, Hill CE, Hines HM (2019). Unsupervised machine learning reveals mimicry complexes in bumblebees occur along a perceptual continuum. Proc. R. Soc. Lond..

[8] Speed MP (1993). Müllerian mimicry and the psychology of predation. Anim. Behav..

[9] Endler JA (1988). Frequency-dependent predation, crypsis and aposematic coloration. Phil. Trans. R. Soc. Lond. B.

[10] Turner JRG, Mallet JLB (1996). Did forest islands drive the diversity of warningly coloured butterflies? Biotic drift and the shifting balance. Philos. Trans. R. Soc. Lond. B.

[11] Lindström L, Alatalo RV, Lyytinen A, Mappes J (2001). Strong antiapostatic selection against novel rare aposematic prey. Proc. Natl. Acad. Sci. U. S. A..

[12] Prudic KL, Skemp AK, Papaj DR (2007). Aposematic coloration, luminance contrast, and the benefits of conspicuousness. Behav. Ecol..

[13] Aronsson M, Gamberale-Stille G (2009). Importance of internal pattern contrast and contrast against the background in aposematic signals. Behav. Ecol..

[14] Guilford T (1986). How do “warning colours” work? conspicuousness may reduce recognition errors in experienced predators. Anim. Behav..

[15] Gamberale-Stille G (2001). Benefit by contrast: an experiment with live aposematic prey. Behav. Ecol..

[16] Arenas LM, Walter D, Stevens M (2015). Signal honesty and predation risk among a closely related group of aposematic species. Sci. Rep..

[17] Arenas LM, Troscianko J, Stevens M (2014). Color contrast and stability as key elements for effective warning signals. Front. Ecol. Evol..

[18] Ruxton GD, Speed MP, Broom M (2009). Identifying the ecological conditions that select for intermediate levels of aposematic signaling. Evol. Ecol..

[19] Nokelainen O, Valkonen J, Lindstedt C, Mappes J (2013). Changes in predator community structure shifts the efficacy of two warning signals in Arctiid moths. J. Anim. Ecol..

[20] Fabricant SA, Herberstein ME (2015). Hidden in plain orange: aposematic coloration is cryptic to a colorblind insect predator. Behav. Ecol..

[21] Bocek M, Kusy D, Motyka M, Bocak L (2019). Persistence of multiple patterns and intraspecific polymorphism in multi-species Müllerian communities of net-winged beetles. Front. Zool..

[22] Masek M, Motyka M, Kusy D, Bocek M, Li Y, Bocak L (2018). Molecular phylogeny, diversity and zoogeography of net-winged beetles (Coleoptera: Lycidae). Insects.

[23] Moore BP, Brown WV (1981). Identification of warning odour components, bitter principles and antifeedants in an aposematic beetle: *Metriorrhynchus rhipidius* (Coleoptera: Lycidae). Ins. Biochem..

[24] Guilford T, Nicol C, Rotschild M, Moore BP (1987). The biological roles of pyrazines: evidence for a warning odour function. Biol. J. Linn. Soc..

[25] Linsley EG, Eisner T, Klots AB (1961). Mimetic assemblages of sibling species of lycid beetles. Evolution.

[26] Endler JA (1993). The color of light in forests and its implications. Ecol. Monogr..

[27] Lingafelter SW (2013). Hispaniolan Hemilophini (Coleoptera, Cerambycidae, Lamiinae). ZooKeys.

[28] Lamas G (2004). Atlas of Neotropical Lepidoptera. Checklist: Part 4A Hesperioidea—Papiionoidea.

[29] Sklenarova K, Chesters D, Bocak L (2013). Phylogeography of poorly dispersing net-winged beetles: a role of drifting india in the origin of afrotropical and oriental fauna. PLoS ONE.

[30] Bocak L, Kundrata R, Andújar FC, Vogler AP (2016). The discovery of Iberobaeniidae (Coleoptera: Elateroidea): a new family of beetles from Spain, with immatures detected by environmental DNA sequencing. Proc. R. Soc. Lond. B.

[31] Hall R (2002). Cenozoic geological and plate tectonic evolution of SE Asia and the SW Pacific: computer-based reconstructions, model and animations. J. Asian Earth Sci..

[32] Merckx VSFT (2015). Evolution of endemism on a young tropical mountain. Nature.

[33] Kazantsev SV (2018). New and little known net-winged beetles (Coleoptera: Lycidae) from the Crocker Range Mountains, Sabah, East Malaysia. Russ. Entomol. J..

[34] Motyka M (2019). Male identification, generic classification and sexual dimorphism of *Micronychus pardus* (Kazantsev, 2018) comb nov (Coleoptera: Lycidae: Calochrominae). Zootaxa.

[35] Aronsson M, Gamberale-Stille G (2013). Evidence of signaling benefits to contrasting internal color boundaries in warning coloration. Behav. Ecol..

[36] Wilson J, Williams K, Forister M (2012). Repeated evolution in overlapping mimicry rings among North American velvet ants. Nat. Commun..

[37] Jiruskova A, Motyka M, Bocek M, Bocak L (2019). The Malacca Strait separates distinct faunas of poorly-flying *Cautires* net-winged beetles. PeerJ.

[38] Malohlava V, Bocak L (2010). Evidence of extreme habitat stability in a Southeast Asian biodiversity hotspot based on the evolutionary analysis of neotenic net-winged beetles. Mol. Ecol..

[39] Nater A (2017). Morphometric, behavioral, and genomic evidence for a new orangutan species. Curr. Biol..

[40] Willmott KR, Willmott JCR, Elias M, Jiggins CD (2017). Maintaining mimicry diversity: optimal warning colour patterns differ among microhabitats in Amazonian clearwing butterflies. Proc. R. Soc. B.

[41] Gompert Z, Willmott KR, Elias M (2011). Heterogeneity in predator micro-habitat use and the maintenance of Müllerian mimetic diversity. J. Theor. Biol..

[42] Eisner T (2008). Defensive chemistry of lycid beetles and of mimetic cerambycid beetles that feed on them. Chemoecology.

[43] Bocak L, Li Y, Ellenberger S (2019). The discovery of *Burmolycus compactus* gen. et sp. Nov. from the mid-Cretaceous of Myanmar provides the evidence for early diversification of net-winged beetles (Coleoptera, Lycidae). Cret. Res..

[44] Motyka M, Kampova L, Bocak L (2018). Phylogeny and evolution of Müllerian mimicry in aposematic *Dilophotes*: evidence for advergence and size-constraints in evolution of mimetic sexual dimorphism. Sci. Rep..

[45] Guilford T (1988). The evolution of conspicuous coloration. Am. Nat..

[46] Alatalo RV, Mappes J (1996). Tracking the evolution of warning signals. Nature.

[47] Speed MP (2000). Warning signals, receiver psychology and predator memory. Anim. Behav..

[48] Mappes J, Alatalo RV (1997). Batesian mimicry and signal accuracy. Evolution.

[49] Motyka M, Masek M, Bocak L (2017). Congruence between morphology and molecular phylogeny: the reclassification of Calochromini (Coleoptera: Lycidae) and their dispersal history. Zool. J. Linn. Soc..

[50] Tiana L, Rahmana SR, Ezrayb BD, Franzini L, Strangec JP, Lhommea P, Hinesa HMA (2019). Homeotic shift late in development drives mimetic color variation in a bumble bee. Proc. Nat. Acad. Sci. USA.

[51] Lewis JJ (2019). Parallel evolution of ancient, pleiotropic enhancers underlies butterfly wing pattern mimicry. Proc. Natl. Acad. Sci. USA.

[52] Aubier TG, Sherratt TN (2015). Diversity in Müllerian mimicry: The optimal predator sampling strategy explains both local and regional polymorphism in prey. Evolution.

[53] Endler JA, Mappes J (2004). Predator mixes and the conspicuousness of aposematic signals. Am. Nat..

[54] Ree RH (2005). Detecting the historical signature of key innovations using stochastic models of character evolution and cladogenesis. Evolution.

[55] Keller SR, Taylor DR (2008). History, chance and adaptation during biological invasion: separating stochastic phenotypic evolution from response to selection. Ecol. Lett..

[56] Bocak L, Yagi T (2010). Evolution of mimicry patterns in *Metriorrhynchus* (Coleoptera: Lycidae): the history of dispersal and speciation in southeast Asia. Evolution.

[57] Kusy D, Motyka M, Bocek M, Masek M, Bocak L (2019). Phylogenomic analysis resolves the relationships among net-winged beetles (Coleoptera: Lycidae) and reveals the parallel evolution of morphological traits. Syst. Ent..

[58] Katoh K, Standley DM (2013). MAFFT multiple sequence alignment software version 7: improvements in performance and usability. Mol. Biol. Evol..

[59] Nguyen LT, Schmidt HA, von Haeseler A, Minh BQ (2015). IQ-TREE: a fast and effective stochastic algorithm for estimating maximum-likelihood phylogenies. Mol. Biol. Evol..

[60] Kalyaanamoorthy S, Minh BQ, Wong TKF, von Haeseler A, Jermiin LS (2017). ModelFinder: fast model selection for accurate phylogenetic estimates. Nat. Methods.

[61] Lanfear R, Frandsen PB, Wright AM, Senfeld T, Calcott B (2016). PartitionFinder 2: new methods for selecting partitioned models of evolution for molecular and morphological phylogenetic analyses. Mol. Biol. Evol..

[62] Drummond AJ, Suchard MA, Xie D, Rambaut A (2012). Bayesian phylogenetics with BEAUti and the BEAST 1.7. Mol. Biol. Evol..

[63] Papadopoulou A, Anastasiou I, Vogler AP (2010). Revisiting the insect mitochondrial molecular clock: the mid-aegean trench calibration. Mol. Biol. Evol..

[64] Rambaut A, Drummond AJ, Xie D, Baele G, Suchard MA (2018). Posterior summarisation in Bayesian phylogenetics using Tracer 1.7. Syst. Biol..

