# Supplementary material for: Interactions in multi-pattern Müllerian communities support origins of new patterns, false structures, imperfect resemblance and mimetic sexual dimorphism
Source: Sci Rep. 2020 Jul 8;10:11193. doi: 10.1038/s41598-020-68027-w (PMC7343875; doi:10.1038/s41598-020-68027-w)
Supplement: Supplementary file 1 — Supplementary information [file 41598_2020_68027_MOESM1_ESM.pdf]

Interactions in multi-pattern Müllerian communities support origins of new patterns, false structures, imperfect resemblance and mimetic sexual dimorphism

Michal Motyka, Matej Bocek, Dominik Kusy & Ladislav Bocak

Laboratory of Diversity and Molecular Evolution, Palacky University, 17. Listopadu 50, 771 46, Olomouc, Czech Republic

The list of Supplementary files

Table S1. The list of Metriorrhynchini samples included in the analysis with geographic origins, voucher, and GenBank accession numbers.

Table S2. Primers and conditions used for PCR amplifications.

Table S3. Characteristics of concatenated supermatrices and used models of the DNA evolution.

Table S4. The list of genera and their distribution.

Table S5. The list of Bornean species and their occurrence.

Table S6. The list of individuals collected on Borneo and their distribution.

Table S7. The list of Calochromini samples included in the analysis with geographic origins, voucher, and GenBank accession numbers.

Figure S1. Maximum likelihood tree recovered by the analysis of the Metriorrhynchini full dataset.

Figure S2. Maximum likelihood tree recovered by the analysis of the Calochromini full dataset.

Figure S3. Maximum likelihood tree recovered by the analysis of the Calochromini reduced dataset. **Figure S4.** Time-calibrated, maximum clade credibility tree computed in BEAST from the reduced dataset with the topology constrained to the results of the maximum-likelihood analysis, the full resolution version of the Fig. 3A.

Figure S5. Phylogenetic tree recovered from the full dataset with designation of aposematic patterns for Bornean samples.

Figure S6. The distribution of the categorized aposematic patterns. Green, Yellow, Brown and Black lines represent species with the reticulate pattern, yellow/black pattern, dark red/brown coloration, and black coloration respectively. Brown pictograms represent samples from a higher elevation, green pictograms represent samples from lowlands.

Table S1. The list of Metriorrhynchini samples included in the analysis with geographic origins, voucher, and GenBank accession numbers.

Voucher	Locality	*rrnL*	*cox1*	*nad5*
000001	Borneo		AB123456	AB123456
000002	Borneo		AB123456	AB123456
000003	Borneo		AB123456	AB123456
000004	Borneo		AB123456	AB123456
000009	Borneo	AB123456	AB123456	AB123456
000010	Sulawesi		AB123456	AB123456
000011	Sulawesi	AB123456	AB123456	AB123456
000013	Borneo		AB123456	AB123456
000014	Borneo		AB123456	AB123456
000015	Borneo		AB123456	AB123456
000016	Borneo		AB123456	AB123456
000017	Sulawesi	AB123456	AB123456	AB123456
000018	Borneo		AB123456	AB123456
000019	Borneo		AB123456	AB123456
000020	Borneo		AB123456	AB123456
000021	Borneo		AB123456	AB123456
000023	Borneo		AB123456	AB123456
000024	Borneo			AB123456
000025	Borneo			AB123456
000026	Borneo	AB123456	AB123456	AB123456
000027	Borneo			AB123456
000028	Borneo		AB123456	
000029	Borneo		AB123456	AB123456
000030	Borneo	AB123456	AB123456	AB123456
000032	Borneo			AB123456
000035	Borneo		AB123456	AB123456
000037	Borneo	AB123456	AB123456	AB123456
000040	Borneo	AB123456	AB123456	AB123456
000043	Borneo	AB123456	AB123456	AB123456
000044	Borneo	AB123456	AB123456	AB123456
000047	Sumatra	AB123456	AB123456	AB123456
000048	Sumatra	AB123456	AB123456	AB123456
000050	Sumatra	AB123456	AB123456	AB123456
000052	Sumatra	AB123456	AB123456	AB123456
000056	Sumatra	AB123456	AB123456	AB123456
000060	Sumatra	AB123456	AB123456	AB123456
000064	Laos	AB123456	AB123456	AB123456
000066	Laos	AB123456	AB123456	AB123456
000068	Borneo	AB123456	AB123456	AB123456
000069	Borneo	AB123456	AB123456	AB123456
000070	Malaysia	AB123456	AB123456	AB123456
000071	Borneo	AB123456	AB123456	AB123456

Voucher	Locality	*rrnL*	*cox1*	*nad5*
000073	Borneo	AB123456	AB123456	AB123456
000074	Borneo	AB123456	AB123456	AB123456
000075	Laos	AB123456	AB123456	AB123456
000078	Borneo			AB123456
000079	Borneo	AB123456	AB123456	AB123456
000080	Borneo	AB123456	AB123456	AB123456
000081	Borneo	AB123456	AB123456	AB123456
000082	Borneo		AB123456	AB123456
000083	Borneo			AB123456
000084	Borneo	AB123456	AB123456	AB123456
000085	Borneo			AB123456
000088	Malaysia	AB123456	AB123456	AB123456
000089	Borneo			AB123456
000090	Borneo	AB123456	AB123456	AB123456
000091	Borneo			AB123456
000092	Borneo			AB123456
000093	Borneo			AB123456
000094	Borneo			AB123456
000104	Borneo	AB123456	AB123456	AB123456
000105	Borneo	AB123456	AB123456	AB123456
000109	Borneo			AB123456
000118	Borneo	AB123456	AB123456	
000119	Borneo			AB123456
000120	Laos	AB123456	AB123456	AB123456
000121	Sumatra	AB123456	AB123456	AB123456
000122	Borneo	AB123456	AB123456	AB123456
000123	Sumatra	AB123456	AB123456	AB123456
000125	Sumatra	AB123456	AB123456	AB123456
000132	Sumatra	AB123456	AB123456	AB123456
000142	Sumatra	AB123456	AB123456	AB123456
000147	India		AB123456	AB123456
000148	Borneo			AB123456
000152	Laos	AB123456	AB123456	AB123456
000153	Laos	AB123456	AB123456	AB123456
000154	Laos	AB123456	AB123456	AB123456
000161	Laos		AB123456	AB123456
000164	Laos	AB123456	AB123456	AB123456
000168	Laos		AB123456	AB123456
000171	Malaysia			AB123456
000172	Malaysia		AB123456	AB123456
000174	Malaysia		AB123456	AB123456
000177	Malaysia	AB123456	AB123456	AB123456

Voucher	Locality	*rrnL*	*cox1*	*nad5*	Voucher	Locality	*rrnL*	*cox1*	*nad5*
000178	Malaysia	AB123456	AB123456	AB123456	000346	Borneo	AB123456	AB123456	AB123456
000179	Borneo			AB123456	000355	Java	AB123456	AB123456	AB123456
000180	Borneo			AB123456	000356	Java		AB123456	AB123456
000181	Borneo			AB123456	000359	Java		AB123456	AB123456
000182	Borneo			AB123456	000366	Palawan	AB123456	AB123456	AB123456
000183	Borneo			AB123456	000372	Australia	AB123456	AB123456	AB123456
000184	Borneo	AB123456	AB123456	AB123456	000373	Australia	AB123456	AB123456	AB123456
000185	Borneo			AB123456	000374	Australia	AB123456	AB123456	AB123456
000186	Borneo			AB123456	000375	Australia	AB123456	AB123456	AB123456
000187	Borneo	AB123456	AB123456	AB123456	000376	Australia	AB123456	AB123456	AB123456
000188	Laos	AB123456	AB123456	AB123456	000378	Australia	AB123456	AB123456	AB123456
000189	Laos	AB123456	AB123456	AB123456	000379	Palawan	AB123456	AB123456	AB123456
000195	South Afri	AB123456	AB123456	AB123456	000380	Palawan			AB123456
000199	Sulawesi	AB123456	AB123456	AB123456	000381	Palawan			AB123456
000205	Sumatra	AB123456	AB123456	AB123456	000382	Palawan			AB123456
000206	Sumatra		AB123456	AB123456	000383	Palawan		AB123456	AB123456
000207	Borneo	AB123456	AB123456	AB123456	000388	Palawan		AB123456	AB123456
000208	Borneo	AB123456	AB123456	AB123456	000390	Palawan		AB123456	AB123456
000217	Japan	AB123456	AB123456	AB123456	000392	Palawan			AB123456
000219	Japan	AB123456	AB123456	AB123456	000393	Palawan		AB123456	AB123456
000221	Japan	AB123456	AB123456	AB123456	000394	Palawan			AB123456
000224	Japan	AB123456	AB123456	AB123456	000395	Palawan	AB123456	AB123456	AB123456
000246	Borneo	AB123456	AB123456	AB123456	000396	Palawan	AB123456	AB123456	AB123456
000248	Sumatra	AB123456	AB123456	AB123456	000399	Palawan			AB123456
000262	Borneo	AB123456	AB123456	AB123456	000402	Palawan	AB123456	AB123456	AB123456
000274	Borneo	AB123456	AB123456	AB123456	000403	Palawan	AB123456	AB123456	AB123456
000290	Laos	AB123456	AB123456	AB123456	000406	Palawan			AB123456
000294	Sumatra	AB123456	AB123456	AB123456	000409	Palawan			AB123456
000295	Sumatra	AB123456	AB123456	AB123456	000411	Palawan	AB123456	AB123456	AB123456
000297	Sumatra	AB123456	AB123456	AB123456	000412	Palawan	AB123456	AB123456	AB123456
000309	Borneo		AB123456	AB123456	000413	Palawan			AB123456
000314	Java	AB123456	AB123456	AB123456	000416	Palawan			AB123456
000315	Java	AB123456	AB123456	AB123456	000418	Palawan			AB123456
000318	Borneo			AB123456	000419	Palawan	AB123456	AB123456	AB123456
000319	Borneo			AB123456	000425	Palawan	AB123456	AB123456	AB123456
000320	Borneo			AB123456	000434	Palawan	AB123456	AB123456	AB123456
000322	Borneo			AB123456	000469	Borneo		AB123456	AB123456
000335	Borneo	AB123456	AB123456	AB123456	000473	Borneo			AB123456
000336	Borneo			AB123456	000478	Borneo		AB123456	AB123456
000337	Borneo			AB123456	000485	Java		AB123456	AB123456
000339	Borneo	AB123456	AB123456	AB123456	000508	Borneo		AB123456	AB123456
000340	Borneo			AB123456	000526	Laos		AB123456	AB123456
000342	Borneo	AB123456	AB123456	AB123456	000527	Laos		AB123456	AB123456
000343	Borneo			AB123456	000532	Laos		AB123456	AB123456
000345	Borneo			AB123456	000535	Sumatra		AB123456	AB123456

Voucher	Locality	*rrnL*	*cox1*	*nad5*
000537	Sumatra		AB123456	AB123456
000538	Malaysia		AB123456	AB123456
A00017	Taiwan	AB123456	AB123456	AB123456
A00018	Taiwan	AB123456	AB123456	AB123456
A00019	Taiwan	AB123456	AB123456	AB123456
A00021	Taiwan	AB123456	AB123456	
A00022	Madagasc	AB123456	AB123456	AB123456
A00023	Madagasc	AB123456	AB123456	AB123456
A00024	Madagasc	AB123456	AB123456	AB123456
A00025	Madagasc	AB123456	AB123456	AB123456
A00026	Madagascar		AB123456	AB123456
A00027	Madagascar		AB123456	AB123456
A00029	Madagasc	AB123456	AB123456	AB123456
A00030	Madagasc	AB123456	AB123456	AB123456
A00031	Queensla	AB123456	AB123456	AB123456
A00032	Queensla	AB123456	AB123456	AB123456
A00034	Queensla	AB123456	AB123456	AB123456
A00035	Queensla	AB123456	AB123456	AB123456
A00036	Queensla	AB123456	AB123456	AB123456
A00037	Queensla	AB123456	AB123456	AB123456
A00038	Queensla	AB123456	AB123456	AB123456
A00039	Queensla	AB123456	AB123456	AB123456
A00040	Queensla	AB123456	AB123456	AB123456
A00041	Queensla	AB123456	AB123456	AB123456
A00042	Queensland		AB123456	AB123456
A00043	Queensla	AB123456	AB123456	AB123456
A00044	Queensla	AB123456	AB123456	AB123456
A00045	Queensla	AB123456	AB123456	AB123456
A00046	Queensla	AB123456	AB123456	AB123456
A00048	Malaysia	AB123456	AB123456	AB123456
A00049	Malaysia	AB123456	AB123456	AB123456
A00050	Malaysia		AB123456	AB123456
A00052	Philippine	AB123456	AB123456	AB123456
A00053	Philippine	AB123456	AB123456	AB123456
A00054	Philippine	AB123456		AB123456
A00057	Philippines		AB123456	AB123456
A00058	Philippine	AB123456	AB123456	AB123456
A00059	Philippine	AB123456	AB123456	AB123456
A00061	Philippine	AB123456	AB123456	AB123456
A00062	Philippine	AB123456	AB123456	AB123456
A00063	Philippines		AB123456	AB123456
A00064	Philippines		AB123456	AB123456
A00065	Philippines		AB123456	AB123456
A00066	Philippine	AB123456	AB123456	AB123456
A00067	Philippine	AB123456	AB123456	AB123456
Voucher	Locality	*rrnL*	*cox1*	*nad5*
A00068	Philippines	AB123456	AB123456	AB123456
A00069	Philippines	AB123456	AB123456	AB123456
A00070	Philippines	AB123456	AB123456	AB123456
A00071	Philippines	AB123456	AB123456	AB123456
A00073	Philippines	AB123456	AB123456	
A00074	Philippines	AB123456	AB123456	
A00075	Philippines	AB123456	AB123456	AB123456
A00076	Philippines	AB123456	AB123456	AB123456
A00077	Philippines	AB123456	AB123456	AB123456
A00078	Cameroon	AB123456	AB123456	AB123456
A00079	Cameroon	AB123456	AB123456	AB123456
A00080	Cameroon	AB123456	AB123456	AB123456
A00081	Cameroon	AB123456	AB123456	AB123456
A00082	Cameroon	AB123456	AB123456	
A00083	Cameroon	AB123456	AB123456	AB123456
A00084	Cameroon	AB123456	AB123456	AB123456
A00085	Cameroon	AB123456	AB123456	AB123456
A00086	Cameroon	AB123456	AB123456	AB123456
A00087	Cameroon	AB123456	AB123456	AB123456
A00088	Cameroon	AB123456	AB123456	AB123456
A00089	Cameroon	AB123456	AB123456	AB123456
A00090	Cameroon	AB123456	AB123456	AB123456
A00091	Cameroon	AB123456	AB123456	AB123456
A00092	Cameroon	AB123456	AB123456	AB123456
A00093	Cameroon	AB123456	AB123456	AB123456
A00094	Cameroon	AB123456	AB123456	AB123456
A00095	Cameroon	AB123456		AB123456
A00096	Cameroon	AB123456		AB123456
A00097	Cameroon	AB123456	AB123456	AB123456
A00098	Cameroon	AB123456	AB123456	AB123456
A00099	Cameroon	AB123456	AB123456	AB123456
A00100	Cameroon	AB123456	AB123456	AB123456
A00101	Cameroon		AB123456	
A00102	Cameroon		AB123456	AB123456
A00103	Cameroon		AB123456	
A00104	Cameroon	AB123456		AB123456
A00106	Cameroon	AB123456	AB123456	AB123456
A00107	Cameroon		AB123456	
A00109	Cameroon	AB123456	AB123456	AB123456
A00110	Cameroon	AB123456	AB123456	AB123456
A00111	Cameroon	AB123456		AB123456
A00112	Cameroon	AB123456	AB123456	AB123456
A00390	Laos	AB123456	AB123456	AB123456
AJ0003	Malaysia	AB123456	AB123456	AB123456
AJ0028	Malaysia	AB123456	AB123456	AB123456

Voucher	Locality	*rrnL*	*cox1*	*nad5*
AJ0050	Malaysia	AB123456	AB123456	AB123456
AJ0052	Malaysia	AB123456	AB123456	AB123456
AJ0054	Malaysia	AB123456	AB123456	AB123456
L00005	Malaysia	AB123456	AB123456	AB123456
L00006	Malaysia	AB123456	AB123456	AB123456
L00014	Malaysia	AB123456	AB123456	AB123456
L00023	Malaysia	AB123456	AB123456	AB123456
MD0029	Sulawesi		AB123456	AB123456
MD0030	Sulawesi		AB123456	AB123456
MD0033	Sulawesi	AB123456	AB123456	AB123456
MD0034	Sulawesi	AB123456	AB123456	AB123456
MD0036	Sulawesi		AB123456	AB123456
MD0044	Sulawesi	AB123456	AB123456	AB123456
MD0064	Sulawesi	AB123456	AB123456	AB123456
MD0065	Sulawesi		AB123456	AB123456
MD0067	Sulawesi	AB123456	AB123456	AB123456
MD0069	Sulawesi	AB123456	AB123456	AB123456
MD0071	Sulawesi	AB123456	AB123456	AB123456
MD0081	Sulawesi	AB123456	AB123456	AB123456
MD0097	Sulawesi	AB123456	AB123456	AB123456
MD0098	Sulawesi	AB123456	AB123456	AB123456
MD0099	Sulawesi	AB123456	AB123456	AB123456
MD0101	Sulawesi	AB123456	AB123456	AB123456
MD0106	Sulawesi	AB123456	AB123456	AB123456
MD0107	Sulawesi	AB123456	AB123456	AB123456
MD0109	Sulawesi	AB123456	AB123456	AB123456
MD0111	Sulawesi	AB123456	AB123456	AB123456
MD0118	Sulawesi	AB123456	AB123456	AB123456
MD0119	Sulawesi	AB123456	AB123456	AB123456
MD0121	Sulawesi	AB123456	AB123456	AB123456
MD0126	Sulawesi	AB123456	AB123456	AB123456
MD0127	Sulawesi	AB123456	AB123456	AB123456
MD0129	Sulawesi	AB123456	AB123456	AB123456
MD0130	Sulawesi	AB123456	AB123456	AB123456
MD0132	Sulawesi	AB123456	AB123456	AB123456
MD0133	Sulawesi	AB123456	AB123456	AB123456
MD0134	Sulawesi	AB123456	AB123456	AB123456
MD0135	Sulawesi	AB123456	AB123456	AB123456
MD0136	Sulawesi	AB123456	AB123456	AB123456
MD0137	Sulawesi	AB123456	AB123456	AB123456
MD0140	Sulawesi	AB123456	AB123456	AB123456
MD0143	Sulawesi	AB123456	AB123456	AB123456
MD0145	Sulawesi	AB123456	AB123456	AB123456
MD0155	Sulawesi	AB123456	AB123456	AB123456
MD0156	Sulawesi	AB123456	AB123456	AB123456

Voucher	Locality	*rrnL*	*cox1*	*nad5*
MD0157	Sulawesi	AB123456	AB123456	AB123456
MD0169	Sulawesi	AB123456	AB123456	AB123456
MK0747	Borneo	AB123456	AB123456	AB123456
MK0748	Borneo		AB123456	
MK0750	Borneo	AB123456	AB123456	AB123456
MK0757	Borneo	AB123456	AB123456	AB123456
MK0760	Borneo	AB123456	AB123456	
MK0762	Borneo	AB123456	AB123456	AB123456
MK0763	Borneo		AB123456	
MK0765	Borneo		AB123456	
MK0766	Borneo	AB123456	AB123456	AB123456
MK0774	Borneo	AB123456	AB123456	AB123456
MK0776	Borneo	AB123456	AB123456	AB123456
MK0777	Borneo		AB123456	
MK0778	Borneo	AB123456	AB123456	AB123456
MK0780	Borneo	AB123456	AB123456	AB123456
MK0781	Borneo	AB123456	AB123456	AB123456
MK0782	Borneo		AB123456	
MK0784	Borneo		AB123456	
MK0785	Borneo		AB123456	
MK0786	Borneo		AB123456	
MK0787	Borneo		AB123456	
MK0788	Borneo		AB123456	
MK0789	Borneo	AB123456	AB123456	AB123456
MK0791	Borneo		AB123456	AB123456
MK0792	Borneo			
MK0793	Borneo		AB123456	AB123456
MK0797	Borneo	AB123456	AB123456	AB123456
MK0798	Borneo		AB123456	
MK0800	Borneo	AB123456	AB123456	AB123456
MK0801	Borneo	AB123456	AB123456	AB123456
MK0802	Borneo		AB123456	
MK0803	Borneo		AB123456	
MK0804	Borneo		AB123456	
MK0805	Borneo	AB123456	AB123456	AB123456
MK0806	Borneo	AB123456	AB123456	AB123456
MK0807	Borneo	AB123456	AB123456	AB123456
MK0812	Borneo		AB123456	
MK0816	Borneo		AB123456	AB123456
MK0822	Borneo	AB123456		
MK0823	Borneo	AB123456	AB123456	AB123456
MK0828	Borneo		AB123456	
MK0834	Borneo		AB123456	
MK0839	Borneo	AB123456	AB123456	AB123456
MK0842	Borneo	AB123456	AB123456	AB123456

Voucher	Locality	*rrnL*	*cox1*	*nad5*
MK0866	Borneo	AB123456	AB123456	AB123456
MK0867	Borneo		AB123456	
MK0869	Borneo	AB123456	AB123456	AB123456
MK0871	Borneo	AB123456	AB123456	AB123456
MK0873	Borneo		AB123456	AB123456
MK0876	Borneo	AB123456	AB123456	AB123456
MK0879	Borneo		AB123456	
MK0880	Borneo		AB123456	
MK0882	Borneo	AB123456	AB123456	AB123456
MK0888	Borneo		AB123456	
MK0895	Borneo		AB123456	
MK0901	Borneo		AB123456	
MK0915	Borneo		AB123456	
MK0916	Borneo		AB123456	
MK0918	Borneo		AB123456	
MK0919	Borneo	AB123456	AB123456	AB123456
MK0920	Borneo		AB123456	
MK0921	Borneo		AB123456	
MK0922	Borneo	AB123456	AB123456	AB123456
MK0923	Borneo	AB123456	AB123456	AB123456
MK0924	Borneo	AB123456	AB123456	AB123456
MK0930	Borneo	AB123456	AB123456	AB123456
MK0931	Borneo	AB123456	AB123456	AB123456
MK0932	Borneo	AB123456	AB123456	AB123456
MK0933	Borneo		AB123456	
MK0934	Borneo		AB123456	AB123456
MK0935	Borneo	AB123456	AB123456	AB123456
MK0936	Borneo	AB123456	AB123456	AB123456
MK0937	Borneo		AB123456	
MK0938	Borneo			
MK0939	Borneo	AB123456	AB123456	AB123456
MK0940	Borneo	AB123456		AB123456
MK0941	Borneo	AB123456	AB123456	AB123456
MK0942	Borneo		AB123456	
MK0943	Borneo		AB123456	
MK0945	Borneo	AB123456	AB123456	AB123456
MK0946	Borneo	AB123456	AB123456	AB123456
MK0947	Borneo		AB123456	
MK0948	Borneo		AB123456	
MK0949	Borneo		AB123456	
MK0952	Borneo	AB123456	AB123456	AB123456
MK0959	Borneo		AB123456	AB123456
MK0960	Borneo	AB123456	AB123456	AB123456
MK0961	Borneo	AB123456	AB123456	AB123456
MK0963	Borneo		AB123456	

Voucher	Locality	*rrnL*	*cox1*	*nad5*
MK1007	Laos	AB123456	AB123456	AB123456
MK1010	China	AB123456	AB123456	AB123456
MK1011	Malaysia	AB123456	AB123456	AB123456
MK1012	China		AB123456	
MK1013	Philippines		AB123456	AB123456
MK1014	Philippines		AB123456	AB123456
MK1015	Philippines		AB123456	
MK1016	Philippines		AB123456	
MK1020	Borneo	AB123456	AB123456	AB123456
MK1021	Philippines		AB123456	
MK1022	Philippines		AB123456	
MK1028	Malaysia		AB123456	AB123456
MM0006	Malaysia	AB123456	AB123456	
MM0007	Malaysia		AB123456	AB123456
MM0027	Sumatra		AB123456	AB123456
MM0039	Sumatra	AB123456		AB123456
MM0056	Sumatra		AB123456	AB123456
MW0001	Malaysia	AB123456	AB123456	AB123456
MW0002	Sumatra	AB123456	AB123456	AB123456
MW0008	Sumatra	AB123456	AB123456	AB123456
MW0009	Sumatra	AB123456	AB123456	AB123456
MW0010	Malaysia	AB123456		AB123456
MW0011	Malaysia	AB123456		AB123456
MW0012	Malaysia			AB123456
MW0013	Malaysia	AB123456	AB123456	AB123456
MW0014	Sumatra	AB123456		AB123456
MW0022	Sumatra			AB123456
MW0023	Sumatra	AB123456	AB123456	AB123456
MW0029	Sumatra			AB123456
MW0033	Borneo		AB123456	AB123456
VK0007	Malaysia		AB123456	AB123456
VK0011	Malaysia		AB123456	AB123456
VK0012	Malaysia	AB123456	AB123456	AB123456
VK0045	Malaysia	AB123456	AB123456	AB123456
VK0050	Laos		AB123456	AB123456
VK0069	Sumatra		AB123456	AB123456
VK0070	Sumatra	AB123456	AB123456	AB123456
VK0075	Japan	AB123456	AB123456	AB123456
VK0081	Sumatra	AB123456	AB123456	AB123456
VK0087	Malaysia	AB123456	AB123456	AB123456
VK0088	Malaysia		AB123456	AB123456
VK0090	Japan		AB123456	AB123456
VK0093	Japan		AB123456	AB123456
VK0098	Sumatra	AB123456	AB123456	AB123456
VK0100	Borneo	AB123456	AB123456	AB123456

Voucher	Locality	*rrnL*	*cox1*	*nad5*
MK0969	Borneo		AB123456	
VK0126	Malaysia		AB123456	AB123456
VK0127	Malaysia		AB123456	AB123456
VK0128	Malaysia	AB123456	AB123456	AB123456
VK0153	Japan	AB123456	AB123456	AB123456
VK0158	Malaysia	AB123456	AB123456	AB123456
VK0160	Malaysia	AB123456	AB123456	AB123456
VK0162	Sumatra		AB123456	AB123456
VK0178	Sumatra		AB123456	AB123456
VK0180	Malaysia		AB123456	AB123456
VK0183	Sumatra		AB123456	AB123456
VK0188	Malaysia		AB123456	AB123456
VK0256	Malaysia	AB123456	AB123456	AB123456
VK0261	Malaysia	AB123456	AB123456	AB123456
VK0262	Malaysia		AB123456	AB123456
VK0276	Malaysia		AB123456	AB123456
VK0315	Malaysia		AB123456	AB123456
VK0320	Malaysia	AB123456	AB123456	AB123456
VK0404	Sumatra		AB123456	AB123456
VK0407	Sumatra		AB123456	AB123456
VK0422	Malaysia		AB123456	AB123456
VK0427	Sumatra	AB123456	AB123456	AB123456
VK0439	Sumatra	AB123456	AB123456	AB123456
VK0441	Sumatra	AB123456	AB123456	AB123456
VK0446	Sumatra		AB123456	AB123456
VK0447	Sumatra		AB123456	AB123456
VK0451	Malaysia	AB123456	AB123456	AB123456
VK0452	Malaysia		AB123456	AB123456
VK0472	Borneo			
VK0486	Japan	AB123456	AB123456	AB123456
VK0488	Japan		AB123456	AB123456
VK0493	Japan		AB123456	AB123456
VK0529	Malaysia	AB123456	AB123456	AB123456
VK0550	Laos		AB123456	AB123456
VK0551	Laos	AB123456	AB123456	AB123456
VK0554	Malaysia		AB123456	AB123456
VK0603	Cambodia	AB123456	AB123456	AB123456
VK0625	Malaysia		AB123456	AB123456
VK0627	Malaysia		AB123456	AB123456
VK0743	China	AB123456	AB123456	AB123456

Table S2. Primers and conditions used for PCR amplifications.

rrnL mtDNA		
16a		CGC CTG TTT AAC AAA AAC AT
ND1A	27	GGT CCC TTA CGA ATT TGA ATA TAT CCT
cox1 mtDNA		
Marcy	27	TAR TTC RTA TGW RCA ATA YCA YTG RTG
JerryN	23	CAA CAY YTA TTT TGR TTY TTT GG
MarcyN	24	TTC RTA WGT TCA RTA TCA TTG RTG
nad5-tRNAs mtDNA		
OF1	29	CCT ACT CCT GTT TCT GCT TTA GTT CAT TC
R6	29	GAA ACG AAA AAT CGT ATT TAA TTT CGA CT
R2M	29	AAT TGA ASC CAA AAA GAG GTA TAT CAC TG

16S-41 *rrnL*

96°C – 2 min

96°C - 30 sec, 41°C – 30 sec, 72°C – 60 sec (40 cycles)

72°C – 10 min, 4°C – 1h, 15°C – infinity, pause

40 cycles

LONG45 – *cox1*, *nad5*

94°C– 1min
94°C – 1 min, 45°C – 1 min, 72°C – 2 min (40 cycles)
72°C – 10min
4°C – 1h, 15°C – infinity, pause

bioline 145

94°C – 2min
94°C – 30 sec, 45°C – 30 sec, 72°C – 1:45 min
72°C – 10 min, 4°C–1h, 15°C – infinity, pause

Table S3. Characteristics of concatenated supermatrices and used models of the DNA evolution (descriptions of columns abbreviation listed bellow tables).

Matrix Metriorrhynchinae: 507 taxa with 8 partitions and 3277 total sites.

Fragment	Seq	Site	Infor	Invar	Model
cox1 mtDNA	444	782	444	300	GTR+F+I+G4
nad1 mtDNA	289	123	77	22	TPM3u+F+I+G4
tRNAs (*nad5*)	442	305	173	84	TIM2+F+I+G4
tRNA-Leu	421	60	18	39	TIM2+F+I+G4
nad5 mtDNA	450	1014	758	181	GTR+F+I+G4
cox2 mtDNA	416	257	186	52	TN+F+I+G4
tRNA-Leu (*rrnL*)	289	71	22	36	HKY+F+G4
rrnL rRNA	313	665	333	239	GTR+F+I+G4

Matrix Metriorrhynchinae reduced dataset: 259 taxa with 8 partitions and 3232 total sites.

Fragment	Seq	Site	Infor	Invar	Model
tRNA-Leu	236	60	16	40	TIM2+F+I+G4
*tRNA*s (nad5)	250	275	156	71	TIM3+F+I+G4
cox1 mtDNA	259	782	438	301	GTR+F+I+G4
nad5 mtDNA	254	1014	746	199	GTR+F+I+G4
nad1 mtDNA	203	123	76	26	TIM2+F+I+G4
cox2 mtDNA	235	257	180	55	TN+F+I+G4
rrnL rRNA	225	650	314	240	GTR+F+I+G4
tRNA-Leu (*rrnL*)	203	71	19	40	TIM+F+I+G4

Matrix Calochromini full dataset: 72 taxa with 8 partitions and 3162 total sites.

Fragment	Seqs	Sites	Infor	Invar	Model
tRNA-Leu	46	59	6	49	TN+F+G4
tRNAs (nad5)	70	245	87	104	TN+F+I+G4
nad5 mtDNA	70	1014	658	274	K3Pu+F+I+G4
nad1 mtDNA	37	124	58	50	TIM2+F+I+G4
cox1 mtDNA	70	782	363	354	TPM3u+F+G4
cox2 mtDNA	45	260	104	144	GTR+F+I+G4
tRNA-Leu (*rrnL*)	61	612	241	305	GTR+F+I+G4
rrnL rRNA	38	66	7	55	GTR+F+I+G4

Matrix Calochromini reduced dataset: 55 taxa with 8 partitions and 3158 total sites.

Fragment	Seq	Site	Infor	Invar	Model
tRNA-Leu	34	59	5	48	TIM2+F+I+G4
cox1 mtDNA	55	782	362	355	GTR+F+I+G4
nad5 mtDNA	52	1014	647	277	GTR+F+I+G4
cox2 mtDNA	33	257	100	129	TIM2+F+I+G4
nad1 mtDNA	27	124	54	47	K3Pu+F+G4
tRNAs (*nad5*)	52	244	86	108	TPM3+F+G4
tRNA-Leu (*rrnL*)	27	66	6	54	TN+F+G4
rrnL rRNA	48	612	239	313	GTR+F+I+G4

Abbreviations:
Seq: Number of sequences
Site: Number of bases
Infor: Number of parsimony-informative sites
Invar: Number of invariant sites
Model: Best-fit model according to BIC using ModelFinder.

Table S4. The list of genera and their distribution

		Austral. *	Afrotr. **	Palear. ***	Oriental	Bor.	Mal.	Sum.	Java	I-B.	Pal.	Phi.	Ind.
Xylometanoeus	11	0	0	3	8	4	1	1	0	1	0	1	0
Metanoeus	21	0	0	1	20	7	3	6	1	1	2	1	0
Metanoeina	32	0	0	4	28	11	4	7	1	2	2	2	0
Sulabanus	11	8	0	0	3	0	0	0	0	0	0	3	0
Trichalus	1	1	0	0	0	0	0	0	0	0	0	0	0
Microtrichalus	6	3	0	0	3	1	0	0	0	0	1	2	0
Synchonnus	1	1	0	0	0	0	0	0	0	0	0	0	0
Leptotrichalus	7	0	0	0	7	2	0	0	0	0	2	3	0
Wakarumbia	16	16	0	0	0	0	0	0	0	0	0	0	0
Cautiromimus	2	1	0	0	1	0	0	0	0	0	1	0	0
Metriorrh. indet.	1	1	0	0	0	0	0	0	0	0	0	0	0
Broxylus	5	5	0	0	0	0	0	0	0	0	0	0	0
Porrostoma	5	5	0	0	0	0	0	0	0	0	0	0	0
Metriorrhynchus	11	7	0	0	4	2	2	0	0	1	1	0	0
Metriorrhynchina	66	48	0	0	18	5	2	0	0	1	5	8	0
Xylobanus	20	1	0	2	17	4	3	0	1	6	4	1	0
Cautires	123	0	27	9	87	33	17	19	2	9	6	2	1
Cautirina	143	1	27	11	104	37	20	19	3	15	10	3	1
Metriorrhynchini	241	49	27	15	150	53	26	26	4	18	17	13	1

* Australian region- the islands east of the Wallace line (Sulawesi, Moluccas, New Guinea), continental Australia
** Continental Sub-Saharan Africa, Madagascar
***Palearctic region- continental China, Japan and Taiwan

Table S5. The list of Bornean species and their distribution.

*occurs also in Malaysia	Borneo lowlands								Sabah			# of localities	
	Kalimantan			Sabah					Highlands			lowl.	highl.
Xylometanoeus	Loksado	Balikpapan	M. Teweh	Poring	Emas-low	Sapulut	Punggul	Tenom	Emas	Kundasang	Mesilau		
Xlm02							1					1	
Xlm03		1										1	
Xlm04					1	1						2	
Xlm11									1	1	1		3
Metanoeus													
Meta02				1								1	
Meta04				1								1	
Meta06				1								1	
Meta07										1			1
Meta10			1									1	
Meta14			1									1	
Meta20									1	1			2
Micritrichalus													
Mtr05					1							1	
Leptotrichalus													
Lep01			1									1	
Lep07					1							1	
Metriorrhynchus													
Mtr10*	1		1	1								3	
Xylobanus													
Xyl02			1									1	
Xyl11*				1			1					2	
Xyl17				1								1	
Xyl20	1			1								2	
Cautires													
Cau06*				1				1				2	
Cau31									1				1
Cau32										1			1
Cau34										1			1
Cau36				1								1	
Cau37	1											1	
Cau38				1								1	
Cau43									1				1
Cau44									1				1
Cau45									1	1			2
Cau49			1									1	
Cau54				1	1							2	
Cau57									1				1
Cau59				1								1	
Cau62	1											1	
Cau67									1	1			2
Cau78									1	1			2
Cau86													1
Cau90	1											1	
Cau91			1									1	
Cau92									1				1
Cau93									1				1
Cau94									1				1
Cau96									1	1			2
Cau99	1											1	
Cau103				1								1	
Cau106									1				1
Cau109	1											1	
Cau116			1									1	
Cau119	1			1								2	

Cau120						1						1
Cau122				1								1
Cau123	1											1
Number of species	9	1	8	15	4	2	2	1	14	9	1	

Table S6. The list of individuals collected on Borneo and their distribution.

		Locality	coordinates	elevation	Borneo lowlands Kalimantan			Sabah					Sabah Highlands			# of localities lowl.	highl.
Xylometanoeus		(all Borneo)			Loksado	Balikpapan	M. Teweh	Poring	Emas-low	Sapulut	Punggul	Tenom	Emas	Kundasang	Mesilau		
Xlm02	LB0071	Sabah, Batu Pungg	4°36.2'N, 116°35.2'E	350m							1					1	
Xlm03	MK1020	Timur Prov., Balikp	01°13´4´S, 116°22.6E	66m		1										1	
Xlm04	MK0924	Sabah, Gn. Emas,	5°48.1'N, 116°14.8'E	650m					1							2	
	LB0478	Sabah, Sapulut	4°36.3'N, 116°49.5'E	500m						1							
Xlm11	LB0179	Sabah, Gn.Emas	5°49.5'N, 116°19.5'E	1650m									1				3
	LB0180	Sabah, Gn.Emas	5°49.5'N, 116°19.5'E	1650m									1				
	LB0181	Sabah, Gn.Emas	5°49.5'N, 116°19.5'E	1650m									1				
	LB0182	Sabah, Gn.Emas	5°49.5'N, 116°19.5'E	1650m									1				
	LB0183	Sabah, Gn.Emas	5°49.5'N, 116°19.5'E	1650m									1				
	LB0184	Sabah, Gn.Emas	5°49.5'N, 116°19.5'E	1650m									1				
	LB0185	Sabah, Gn.Emas	5°49.5'N, 116°19.5'E	1650m									1				
	LB0186	Sabah, Gn.Emas	5°49.5'N, 116°19.5'E	1650m									1				
	MK0797	Sabah, Mesilau	6°01.0'N, 116°34.7'E	1650m											1		
	MK0942	Sabah, Kundasang	6°1.5'N, 116°32.7'E,	1700m										1			
	MK0943	Sabah, Kundasang	6°1.5'N, 116°32.7'E,	1700m										1			
	MK0945	Sabah, Kundasang	6°1.5'N, 116°32.7'E,	1700m										1			
	MK0948	Sabah, Kundasang	6°1.5'N, 116°32.7'E,	1700m										1			
	MK0949	Sabah, Kundasang	6°1.5'N, 116°32.7'E,	1700m										1			
	VK0100	Sabah, Gn.Emas	5°49.5'N, 116°19.5'E	1650m										1			
Metanoeus																	
Meta02	MK0816	Sabah, Poring	6.03N, 116.42E	550m				1								1	
Meta04	MK0822	Sabah, Poring	6.03N, 116.42E	550m				1								1	
Meta06	MK0782	Sabah, Poring	6.03N, 116.42E	550m				1								1	
	MK0784	Sabah, Poring	6.03N, 116.42E	550m				1									
	MK0786	Sabah, Poring	6.03N, 116.42E	550m				1									
	MK0789	Sabah, Poring	6.03N, 116.42E	550m				1									
	MK0879	Sabah, Poring	6.03N, 116.42E	550m				1									
Meta07	MK0946	Sabah, Kundasang	6°1.5'N, 116°32.7'E,	1700m										1			1
Meta10	LB0309	Tenggah Prov., M.	1°6.2'S, 115°23.5'E	120m			1									1	
Meta14	LB0105	Tenggah Prov., M.	1°6.2'S, 115°23.5'E	120m			1									1	
Meta20	LB0026	Sabah, Gn. Emas	5°49.5'N, 116°19.5'E	1650m									1				2
	MK0936	Sabah, Kundasang	6°1.5'N, 116°32.7'E	1700m										1			
	MK0937	Sabah, Kundasang	6°1.5'N, 116°32.7'E	1700m										1			
	MW0033	Sabah, Gn. Emas	5°49.5'N, 116°19.5'E	1650m									1				
Micritrichalus																	
Mtr05	LB0023	Selatan Prov., Loks	2°46.9'S, 115°30.8'	350m	1											1	
Leptotrichalus																	
Lep01	LB0208	Tenggah Prov.,M. T	1°6.2'S, 115°23.5'E	120m			1									1	
Lep07	MK0923	Sabah, Gn. Emas, 6	5°48.1'N, 116°14.8'E	650m					1							1	
Metriorrhynchus																	
Mtr09	MK0750	Sabah, Poring	6.03N, 116.42E	550m				1								3	
+ Malay	MK0777	Sabah, Poring	6.03N, 116.42E	550m				1									
	MK0806	Sabah, Poring	6.03N, 116.42E	550m				1									
	MK0807	Sabah, Poring	6.03N, 116.42E	550m				1									
	MK0867	Sabah, Poring	6.03N, 116.42E	550m				1									
	MK0869	Sabah, Poring	6.03N, 116.42E	550m				1									
	LB0001	Selatan Prov., Loks	2°46.9'S, 115°30.8'	350m	1												
	LB0002	Selatan Prov., Loks	2°46.9'S, 115°30.8'	350m	1												

					Lok.	Bal.	Tew	Por	Eml	Sap	Pgg	Ten	Emas	Kund	Mes		
	LB0013	Tenggah Prov., M.	1°6.2'S, 115°23.5'E	120m			1										
	LB0014	Tenggah Prov., M.	1°6.2'S, 115°23.5'E	120m			1										
	LB0016	Tenggah Prov., M.	1°6.2'S, 115°23.5'E	120m			1										
	LB0019	Selatan Prov., Loks	2°46.9'S, 115°30.8'	350m	1												
	LB0020	Selatan Prov., Loks	2°46.9'S, 115°30.8'	350m	1												
	LB0021	Selatan Prov., Loks	2°46.9'S, 115°30.8'	350m	1												
Mtr10	LB0003	Selatan Prov., Loks	2°46.9'S, 115°30.8'	350m	1											3	
+ Malay	LB0004	Selatan Prov., Loks	2°46.9'S, 115°30.8'	350m	1												
	LB0015	Tenggah Prov., M.	1°6.2'S, 115°23.5'E	120m			1										
	LB0018	Selatan Prov., Loks	2°46.9'S, 115°30.8'	350m	1												
	LB0023	Selatan Prov., Loks	2°46.9'S, 115°30.8'	350m	1												
	MK0766	Sabah, Poring	6.03N, 116.42E	550m				1									
	MK0757	Sabah, Poring	6.03N, 116.42E	550m				1									
	MK0785	Sabah, Poring	6.03N, 116.42E	550m				1									
	MK0763	Sabah, Poring	6.03N, 116.42E	550m				1									
	MK0800	Sabah, Poring	6.03N, 116.42E	550m				1									
	MK0803	Sabah, Poring	6.03N, 116.42E	550m				1									
	MK0805	Sabah, Poring	6.03N, 116.42E	550m				1									
Xylobanus																	
Xyl02	LB0274	Tenggah Prov., M.	1°6.2'S, 115°23.5'E	120m			1									1	
Xyl11	LB0469	Sabah, Batu Pungg	4°36.2'N, 116°35.2'E	350m				1			1					2	
	MK0791	Sabah, Poring	6.03N, 116.42E	550m				1									
	MK0793	Sabah, Poring	6.03N, 116.42E	550m				1									
	MK0871	Sabah, Poring	6.03N, 116.42E	550m				1									
	MK0873	Sabah, Poring	6.03N, 116.42E	550m				1									
	MK0895	Sabah, Poring	6.03N, 116.42E	550m				1									
	MK0888	Sabah, Poring	6.03N, 116.42E	550m				1									
	MK0901	Sabah, Poring	6.03N, 116.42E	550m				1									
	MK0959	Sabah, Poring	6.03N, 116.42E	550m				1									
	MK0963	Sabah, Poring	6.03N, 116.42E	550m				1									
Xyl17	MK0828	Sabah, Poring	6.03N, 116.42E	550m				1								1	
	MK0834	Sabah, Poring	6.03N, 116.42E	550m				1									
	MK0839	Sabah, Poring	6.03N, 116.42E	550m				1									
	MK0842	Sabah, Poring	6.03N, 116.42E	550m				1									
	MK0960	Sabah, Poring	6.03N, 116.42E	550m				1									
	MK0969	Sabah, Poring	6.03N, 116.42E	550m				1									
	MK0961	Sabah, Poring	6.03N, 116.42E	550m				1									
Xyl20	MK0778	Sabah, Poring	6.03N, 116.42E	550m				1								2	
	MK0788	Sabah, Poring	6.03N, 116.42E	550m				1									
	MK0798	Sabah, Poring	6.03N, 116.42E	550m				1									
	MK0802	Sabah, Poring	6.03N, 116.42E	550m				1									
	LB0508	Selatan Prov., Loks	2°46.9'S, 115°30.8'	350m	1												
Cautires																	
Cau06	LB0473	Sabah, Tenom	5°8.532'N, 115°55.44	750m								1				2	
+ Malay	MK0781	Sabah, Poring	6.03N, 116.42E	550m				1									
	MK0882	Sabah, Poring	6.03N, 116.42E	550m													
Cau31	LB0069	Sabah, Gn. Emas	5.50°N,116.24°E	1650m									1				1
	LB0083	Sabah, Gn. Emas	5.50°N,116.24°E	1650m									1				
Cau32	MK0932	Sabah, Kundasang	6°1.5'N, 116°32.7'E	1700m										1			1
Cau34	MK0931	Sabah, Kundasang	6°1.5'N, 116°32.7'E	1700m										1			1
Cau36	MK0823	Sabah, Poring	6.03N, 116.42E	550m				1								2	
Cau37	LB0335	Selatan Prov., Loks	2°46.9'S, 115°30.8'	350m													
	LB0336	Selatan Prov., Loks	2°46.9'S, 115°30.8'	350m	1												
Cau38	MK0952	Sabah, Poring	6.03N, 116.42E	550m				1									
Cau43	LB0082	Sabah, Gn. Emas	5.50°N,116.24°E	1650m									1				1
Cau44	LB0068	Sabah, Gn. Emas	5.50°N,116.24°E	1650m									1				1

					Lok.	Bal.	Tew	Por	Eml	Sap	Pgg	Ten	Emas	Kund	Mes		
	LB0085	Sabah, Gn. Emas	5.50°N,116.24°E	1650m									1				
Cau45	LB0030	Sabah, Gn. Emas	5.50°N,116.24°E	1650m									1				2
	MK0916	Sabah, Gn. Emas	5.50°N,116.24°E	1650m									1				
	MK0918	Sabah, Gn. Emas	5.50°N,116.24°E	1650m									1				
	MK0920	Sabah, Gn. Emas	5.50°N,116.24°E	1650m									1				
	MK0930	Sabah, Kundasang	6°1.5'N, 116°32.7'E	1700m										1			
	MK0940	Sabah, Kundasang	6°1.5'N, 116°32.7'E	1700m										1			
Cau49	LB0109	Tenggah Prov., M.	1°6.2'S, 115°23.5'E	120m			1									1	
Cau54	LB0122	Sabah, Gn. Emas, 6	5°48.1'N, 116°14.8'E	650m					1							2	
	MK0801	Sabah, Poring	6.03N, 116.42E	550m				1									
Cau57	LB0029	Sabah, Gn. Emas	5.50°N,116.24°E	1650m									1				1
Cau59	MK0866	Sabah, Poring	6.03N, 116.42E	550m				1								1	
Cau62	LB0043	Selatan Prov., Loks	2°46.9'S, 115°30.8'	350m	1											1	
Cau67	LB00L06	Sabah, Gn. Emas	5.50°N,116.24°E	1650m									1				2
	MK0921	Sabah, Gn. Emas	5.50°N,116.24°E	1650m									1				
	MK0922	Sabah, Gn. Emas	5.50°N,116.24°E	1650m									1				
	MK0933	Sabah, Kundasang	6°1.5'N, 116°32.7'E	1700m										1			
	MK0939	Sabah, Kundasang	6°1.5'N, 116°32.7'E	1700m										1			
	MK0915	Sabah, Gn. Emas	5.50°N,116.24°E	1650m									1				
	LB0024	Sabah, Gn. Emas	5.50°N,116.24°E	1650m									1				
	LB0025	Sabah, Gn. Emas	5.50°N,116.24°E	1650m									1				
	LB0027	Sabah, Gn. Emas	5.50°N,116.24°E	1650m									1				
	LB0078	Sabah, Gn. Emas	5.50°N,116.24°E	1650m									1				
	LB0089	Sabah, Gn. Emas	5.50°N,116.24°E	1650m									1				
	LB0091	Sabah, Gn. Emas	5.50°N,116.24°E	1650m									1				
	LB0093	Sabah, Gn. Emas	5.50°N,116.24°E	1650m									1				
	LB0094	Sabah, Gn. Emas	5.50°N,116.24°E	1650m									1				
	LB0119	Sabah, Gn. Emas	5.50°N,116.24°E	1650m									1				
	LB0207	Sabah, Gn. Emas	5.50°N,116.24°E	1650m									1				
	LB0148	Sabah, Gn. Emas	5.50°N,116.24°E	1650m									1				
Cau78	MK0935	Sabah, Kundasang	6°1.5'N, 116°32.7'E	1700m									1				2
	LB0079	Sabah, Gn. Emas	5.50°N,116.24°E	1650m										1			
Cau86	MK0934	Sabah, Kundasang	6°1.5'N, 116°32.7'E	1700m													1
Cau90	LB0044	Selatan Prov., Loks	2°46.9'S, 115°30.8'	350m	1											1	
Cau91	LB0104	Tenggah Prov., M.	1°6.2'S, 115°23.5'E	120m			1									1	
Cau92	LB0084	Sabah, Gn. Emas	5.50°N,116.24°E	1650m									1				1
Cau93	LB0032	Sabah, Gn. Emas	5.50°N,116.24°E	1650m									1				1
	LB0081	Sabah, Gn. Emas	5.50°N,116.24°E	1650m									1				
Cau94	LB0090	Sabah, Gn. Emas	5.50°N,116.24°E	1650m									1				1
Cau96	LB0028	Sabah, Gn. Emas	5.50°N,116.24°E	1650m									1				2
	LB0080	Sabah, Gn. Emas	5.50°N,116.24°E	1650m									1				
	LB0092	Sabah, Gn. Emas	5.50°N,116.24°E	1650m									1				
	MK0919	Sabah, Gn. Emas	5.50°N,116.24°E	1650m									1				
	MK0941	Sabah, Kundasang	6°1.5'N, 116°32.7'E	1700m										1			
	MK0947	Sabah, Kundasang	6°1.5'N, 116°32.7'E	1700m										1			
Cau99	LB0035	Selatan Prov., Loks	2°46.9'S, 115°30.8'	350m	1											1	
	LB0037	Selatan Prov., Loks	2°46.9'S, 115°30.8'	350m	1												
	LB0040	Selatan Prov., Loks	2°46.9'S, 115°30.8'	350m	1												
	LB0318	Selatan Prov., Loks	2°46.9'S, 115°30.8'	350m	1												
	LB0319	Selatan Prov., Loks	2°46.9'S, 115°30.8'	350m	1												
	LB0320	Selatan Prov., Loks	2°46.9'S, 115°30.8'	350m	1												
	LB0322	Selatan Prov., Loks	2°46.9'S, 115°30.8'	350m	1												
Cau103	MK0747	Sabah, Poring	6.03N, 116.42E	550m				1								1	
	MK0748	Sabah, Poring	6.03N, 116.42E	550m				1									
	MK0762	Sabah, Poring	6.03N, 116.42E	550m				1									

					Lok.	Bal.	Tew	Por	Eml	Sap	Pgg	Ten	Emas	Kund	Mes		
	MK0776	Sabah, Poring	6.03N, 116.42E	550m				1									
	MK0780	Sabah, Poring	6.03N, 116.42E	550m				1									
	MK0787	Sabah, Poring	6.03N, 116.42E	550m				1									
	MK0880	Sabah, Poring	6.03N, 116.42E	550m				1									
	MK0804	Sabah, Poring	6.03N, 116.42E	550m				1									
	MK0876	Sabah, Poring	6.03N, 116.42E	550m				1									
Cau106	LB0074	Sabah, Gn. Emas	5.50°N,116.24°E	1650m									1				1
Cau109	LB0346	Selatan Prov., Loks	2°46.9'S, 115°30.8'	350m	1											1	
Cau116	LB0262	Tenggah Prov., M.	1°6.2'S, 115°23.5'E	120m			1									1	
Cau119	MK0760	Sabah, Poring	6.03N, 116.42E	550m				1								2	
	LB0339	Selatan Prov., Loks	2°46.9'S, 115°30.8'	350m	1												
Cau120	LB0291	Sabah, Tibow	4°36.3'N, 116°49.5'E	500m						1						1	
Cau122	MK0774	Sabah, Poring	6.03N, 116.42E	550m				1								1	
	MK0792	Sabah, Poring	6.03N, 116.42E	550m				1									
	MK0812	Sabah, Poring	6.03N, 116.42E	550m				1									
Cau123	LB0337	Selatan Prov., Loks	2°46.9'S, 115°30.8'	350m	1											1	
	LB0340	Selatan Prov., Loks	2°46.9'S, 115°30.8'	350m	1												
	LB0342	Selatan Prov., Loks	2°46.9'S, 115°30.8'	350m	1												
	LB0343	Selatan Prov., Loks	2°46.9'S, 115°30.8'	350m	1												
	LB0345	Selatan Prov., Loks	2°46.9'S, 115°30.8'	350m	1												
Number of specimens			171		28	1	11	59	3	2	2	1	45	18	1	33	18

Table S7. The list of Calochromini samples included in the analysis with geographic origins, voucher, and GenBank accession numbers.

Outgroups

Voucher	Genus	Species	Locality	*rrnL*	*cox1*	*nad5*
000243	*Plateros*	sp.	Malaya	MT123456	MT123456	MT123456
000244	*Dilophotes*	sp.	Malaya	MT123457	MT123457	MT123457
000348	*Lycoprogenthes*	sp.	Indonesia	MT123458	MT123458	MT123458
000358	*Lycoprogenthes*	sp.	Indonesia	MT123459	MT123459	MT123459
000570	*Dictyoptera*	sp.	Japan	MT123460	MT123460	MT123460
000574	*Pyropterus*	sp.	Japan	MT123461	MT123461	MT123461
000578	*Lopheros*	sp.	Japan	MT123462	MT123462	MT123462
000801	*Lycoprogenthes*	sp.	Japan	MT123463	MT123463	MT123463
001002	*Libnetis*	sp.	Indonesia	MT123464	MT123464	MT123464
A00047	*Plateros*	sp.	Malaya	MT123465	MT123465	MT123465
A00060	*Dilophotes*	sp.	Philippines	MT123466	MT123466	MT123466
000L01	*Duliticola*	sp.	Borneo: Gunung Emas	MT123467	MT123467	MT123467
000L02	*Libnetis*	sp.	Borneo: Gunung Emas	MT123468	MT123468	MT123468
000L03	*Lycus*	sp.	RSA	MT123469	MT123469	MT123469
000L11	*Lyropaeus*	sp.	Borneo: Gunung Emas	MT123470	MT123470	MT123470
000L12	*Dihammatus*	sp.	Borneo: Gunung Emas	MT123471	MT123471	MT123471
000L13	*Plateros*	sp.	Borneo: Gunung Emas	MT123472	MT123472	MT123472
000L15	*Scarelus*	sp.	Borneo: Gunung Emas	MT123473	MT123473	MT123473
000L17	*Lyponia*	sp.	China	MT123474	MT123474	MT123474
000L18	*Macrolycus*	sp.	China	MT123475	MT123475	MT123475
000L25	*Calopteron*	sp.	Equador	MT123476	MT123476	MT123476

Ingroup

Voucher	Genus	Species	Locality	*rrnL*	*cox1*	*nad5*
000033	*Micronychus*	*pardus*	Borneo: Gunung Emas	DQ180986	DQ181208	DQ181362
000124	*Micronychus*	*pardus*	Borneo: Gunung Emas	DQ180987	DQ181209	DQ181363
MT0014	*Micronychus*	*pardus*	Borneo: Gunung Emas	KU495941	KU496072	KU496154
000347	*Micronychus*	sp.	Borneo: Gunung Emas	DQ180994	DQ181216	DQ181370
000400	*Micronychus*	sp.	Malaya	MT123456	MT123457	MT123458
A00477	*Micronychus*	sp.	Malaya	KT751987	KT751669	KT751829
MT0001	*Micronychus*	sp.	China	KU495936	KU496068	KU496184
MT0002	*Micronychus*	sp.	Borneo: Batu Punggul	KU495939	KU496070	KU496152
MT0005	*Micronychus*	sp.	Sumatra	KU495942	KU496073	KU496155
MT0007	*Micronychus*	sp.	Borneo: Sapulut	KU495940	KU496071	KU496153
MT0008	*Micronychus*	sp.	Laos	-	KU496118	KU496163
MT0011	*Micronychus*	sp.	Malaya	KU495938	KU496100	KU496186
MT0012	*Micronychus*	sp.	Borneo: Sapulut	KU495952	KU496082	KU496148
MT0013	*Micronychus*	sp.	India	KU495937	KU496069	KU496185
MT0015	*Micronychus*	sp.	India	-	KU496086	KU496164
MT0016	*Micronychus*	sp.	India	-	KU496087	KU496166
MT0017	*Micronychus*	sp.	India	KU495968	KU496112	KU496172
MT0019	*Micronychus*	sp.	RSA	KU495971	KU496109	KU496202
MT0020	*Micronychus*	sp.	Zambia	KU495973	KU496101	KU496205
MT0021	*Micronychus*	sp.	Zambia	KU495974	KU496102	KU496170
MT0022	*Micronychus*	sp.	RSA	KU495977	KU496115	KU496206
MT0023	*Micronychus*	sp.	Kenya	KU495972	KU496116	KU496171
MT0027	*Micronychus*	sp.	Kenya	KU495975	KU496110	KU496204
MT0028	*Micronychus*	sp.	RSA	-	KU496144	-
MT0029	*Micronychus*	sp.	RSA	-	KU496140	KU496207
MT0031	*Micronychus*	sp.	India	-	KU496088	KU496165
MT0033	*Micronychus*	sp.	Kenya	KU495976	KU496111	KU496203
MT0034	*Micronychus*	sp.	Laos	KU495953	KU496083	KU496149
MT0035	*Micronychus*	sp.	Sumatra	KU495985	KU496105	KU496211
MT0037	*Micronychus*	sp.	Malaya	KU495948	KU496078	KU496161
MT0040	*Micronychus*	sp.	Malaya	KU495954	KU496084	KU496150

MT0043	*Micronychus*	sp.	Malaya	KU495955	KU496085	KU496151
MT0048	*Micronychus*	sp.	China	KU495986	KU496104	KU496210
MT0049	*Micronychus*	sp.	Malaya	KU495945	KU496075	KU496158
MT0050	*Micronychus*	sp.	Laos	KU495949	KU496106	KU496146
MT0051	*Micronychus*	sp.	Laos	KU495946	KU496077	KU496160
MT0052	*Micronychus*	sp.	Laos	KU495991	KU496090	KU496169
MT0055	*Micronychus*	sp.	Malaya	KU495950	KU496107	KU496147
MT0057	*Micronychus*	sp.	Malaya	KU495947	KU496076	-
MT0059	*Micronychus*	sp.	Malaya	KU495943	KU496141	KU496156
MT0060	*Micronychus*	sp.	RSA	-	KU496142	KU496201
MT0063	*Micronychus*	sp.	India	KU495962	KU496093	KU496177
MT0064	*Micronychus*	sp.	India	-	KU496089	KU496167
MT0065	*Micronychus*	sp.	India	KU495966	KU496091	KU496178
MT0066	*Micronychus*	sp.	India	KU495967	KU496119	KU496209
MT0067	*Micronychus*	sp.	India	KU495969	KU496113	KU496208
MT0068	*Micronychus*	sp.	Malaya	KU495944	KU496074	KU496157
MT0070	*Micronychus*	sp.	Laos	KU495951	KU496108	KU496145
MT0071	*Micronychus*	sp.	India	KU495956	KU496103	KU496168
MT0072	*Micronychus*	sp.	India	KU495970	KU496114	KU496173
VK0086	*Micronychus*	sp.	Malaya	MT123456	MT123457	MT123458

Used abbreviation:
RSA – Republic of South Africa

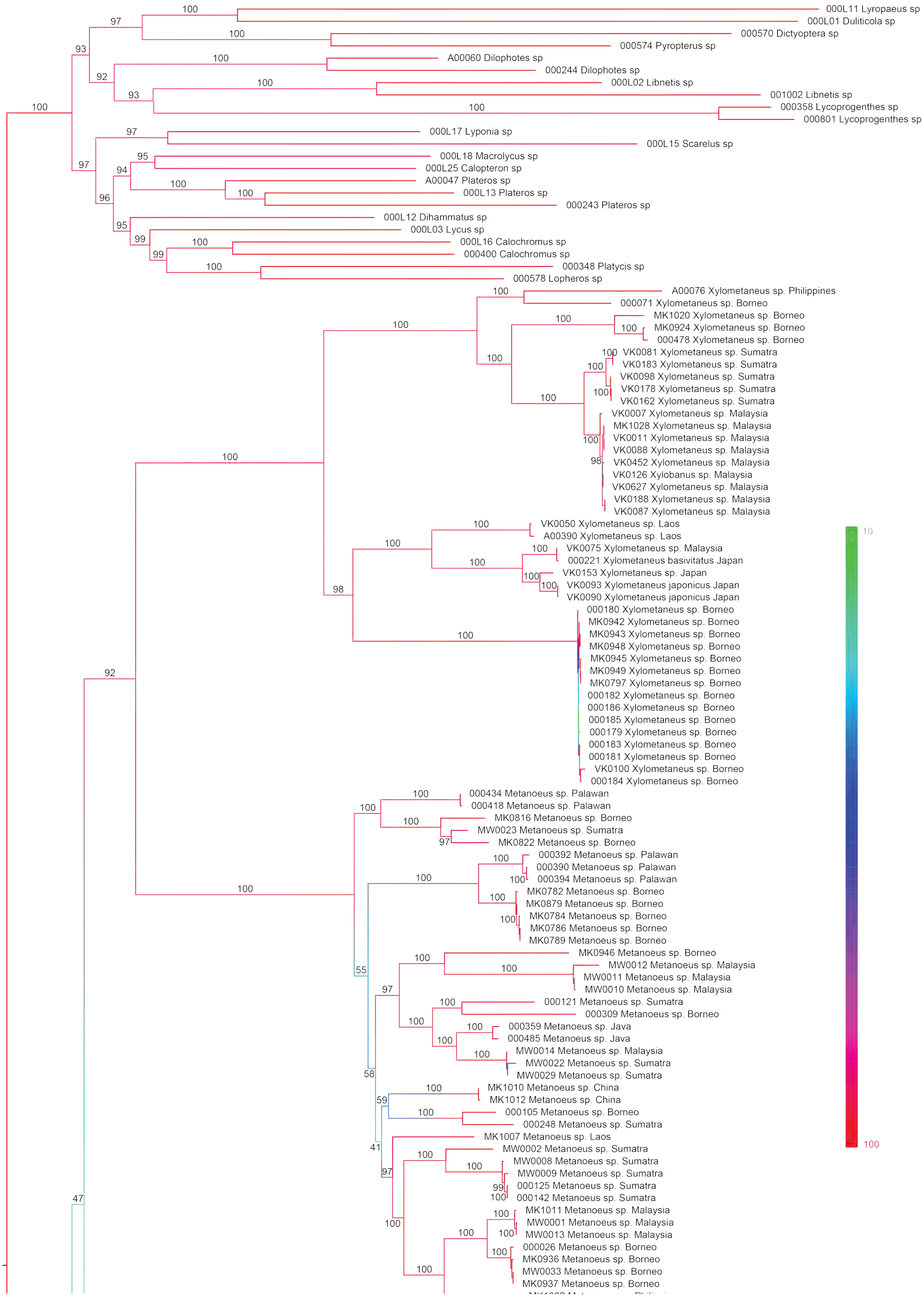

Figure S1. Maximum likelihood tree recovered by the analysis of the Metriorrhynchini full dataset.
Figure continued on next page

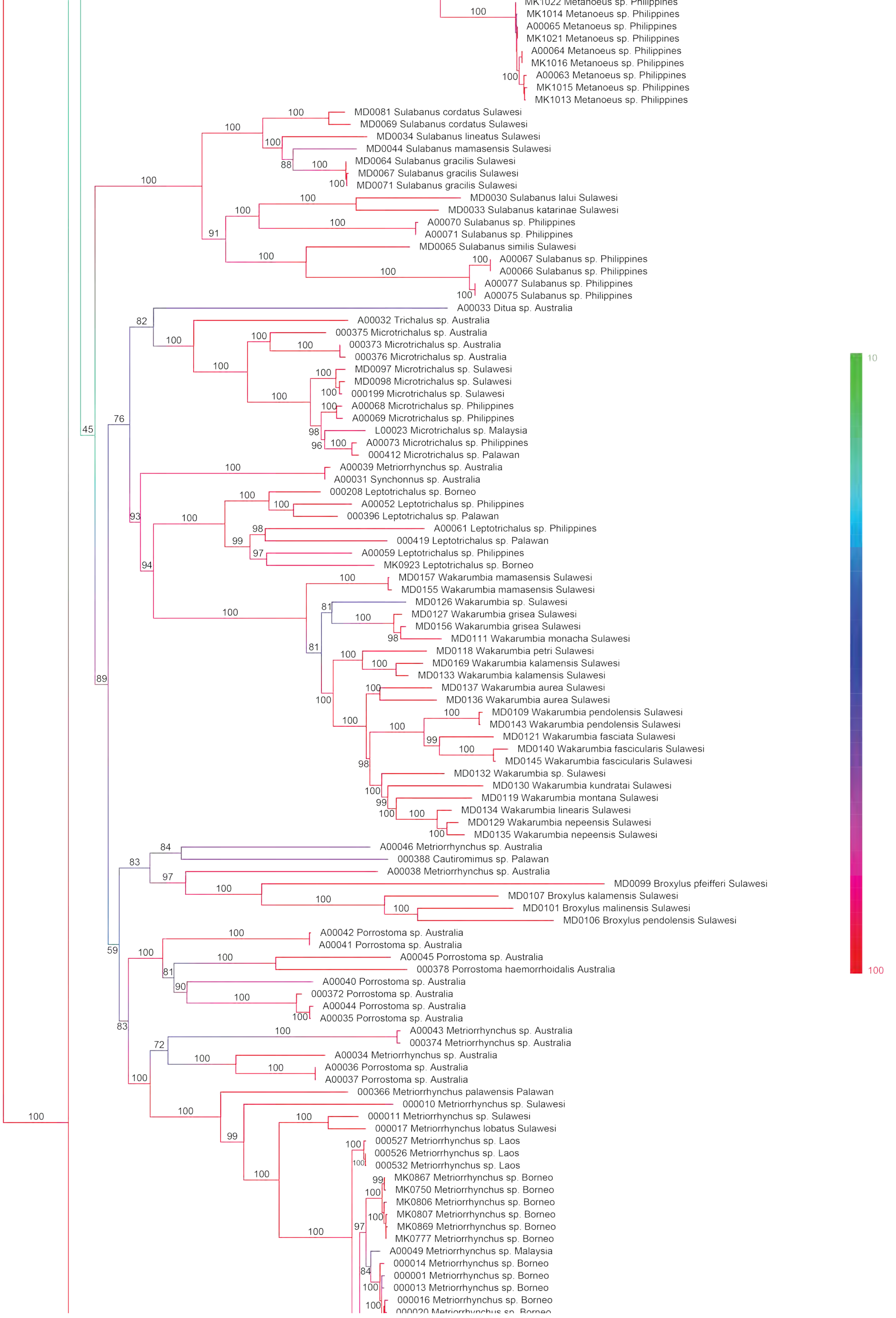

Figure S1. Maximum likelihood tree recovered by the analysis of the Metriorrhynchini full dataset.
Figure continued on next page

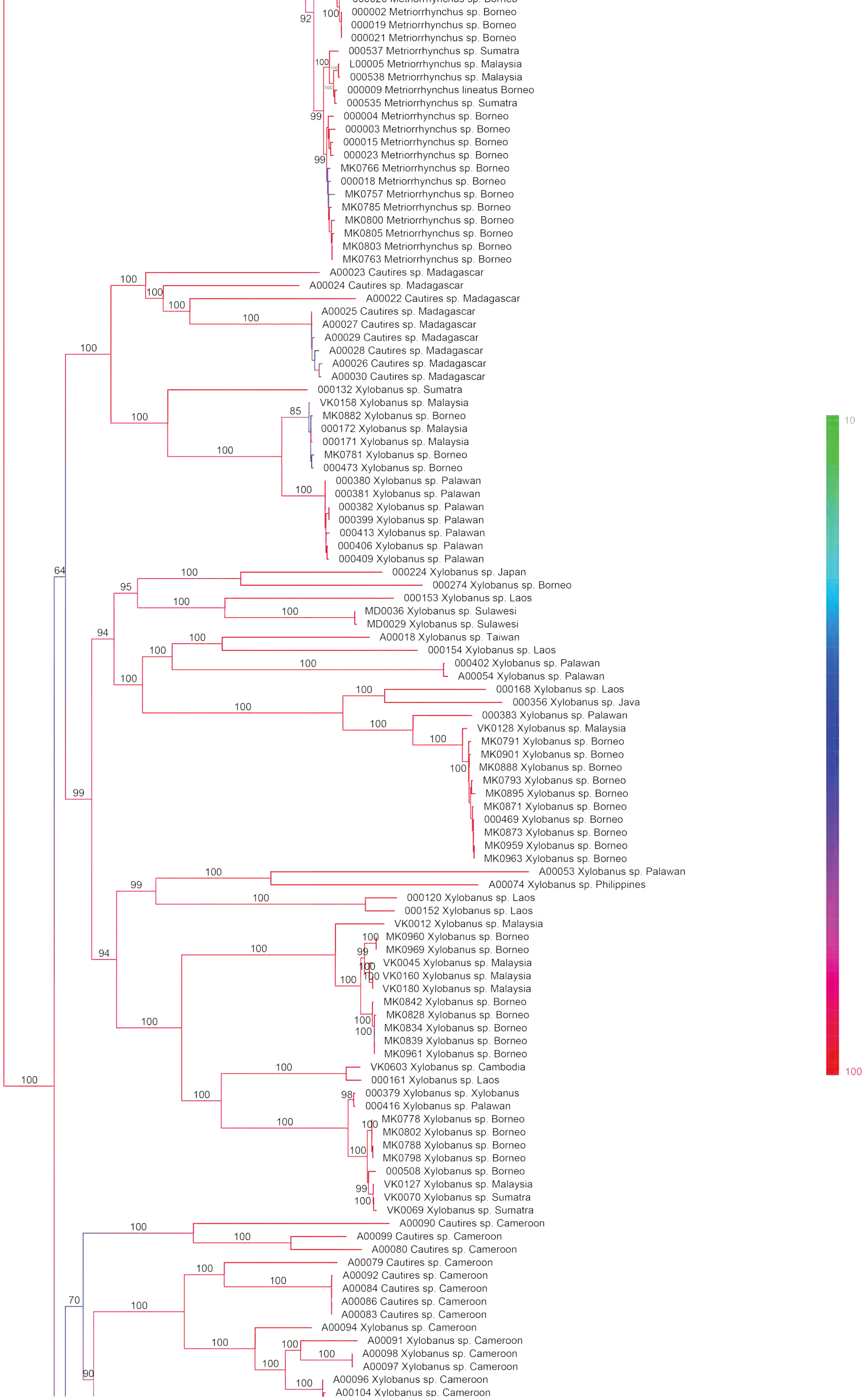

Figure S1. Maximum likelihood tree recovered by the analysis of the Metriorrhynchini full dataset.
Figure continued on next page

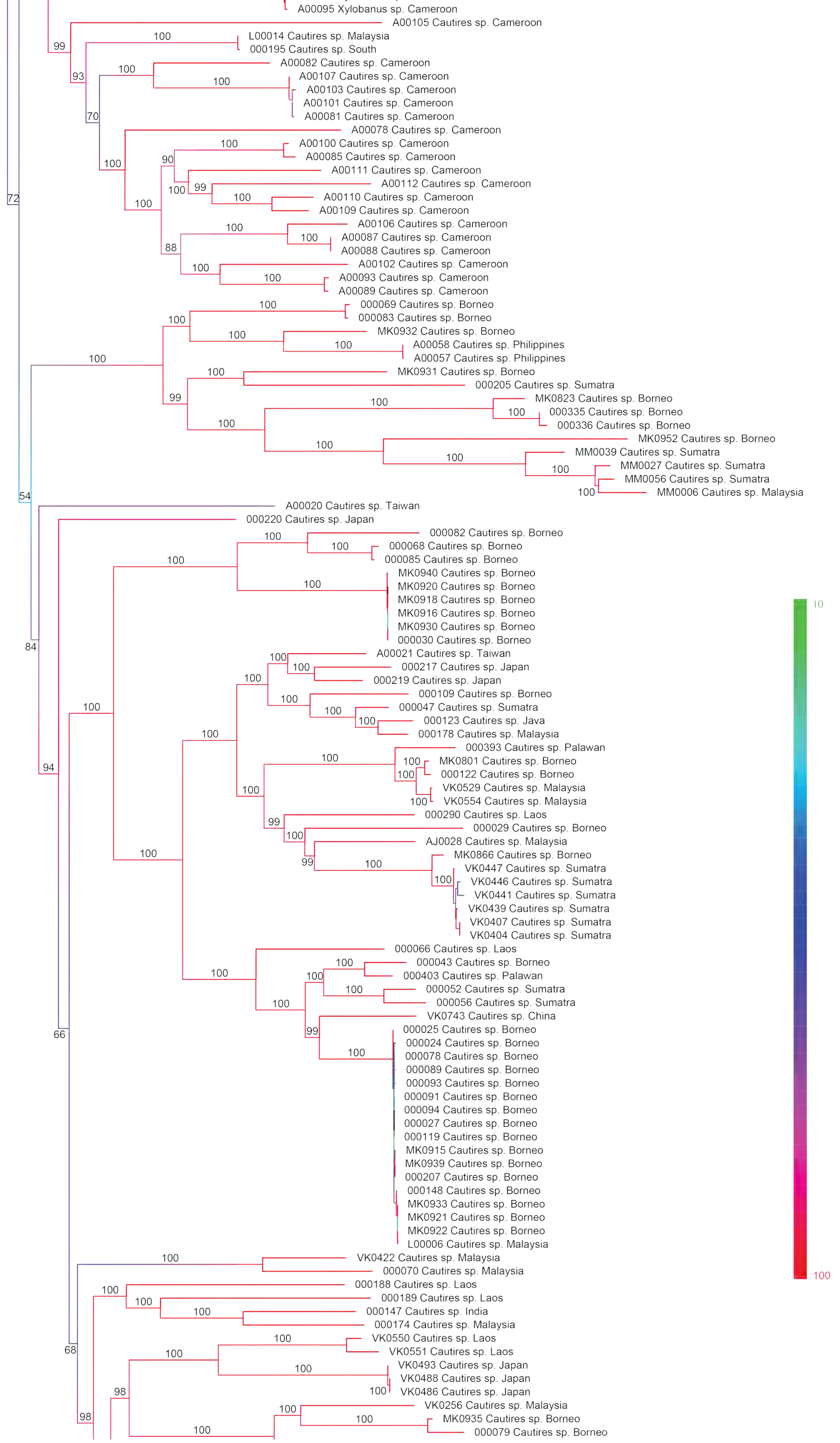

Figure S1. Maximum likelihood tree recovered by the analysis of the Metriorrhynchini full dataset. Figure continued on next page

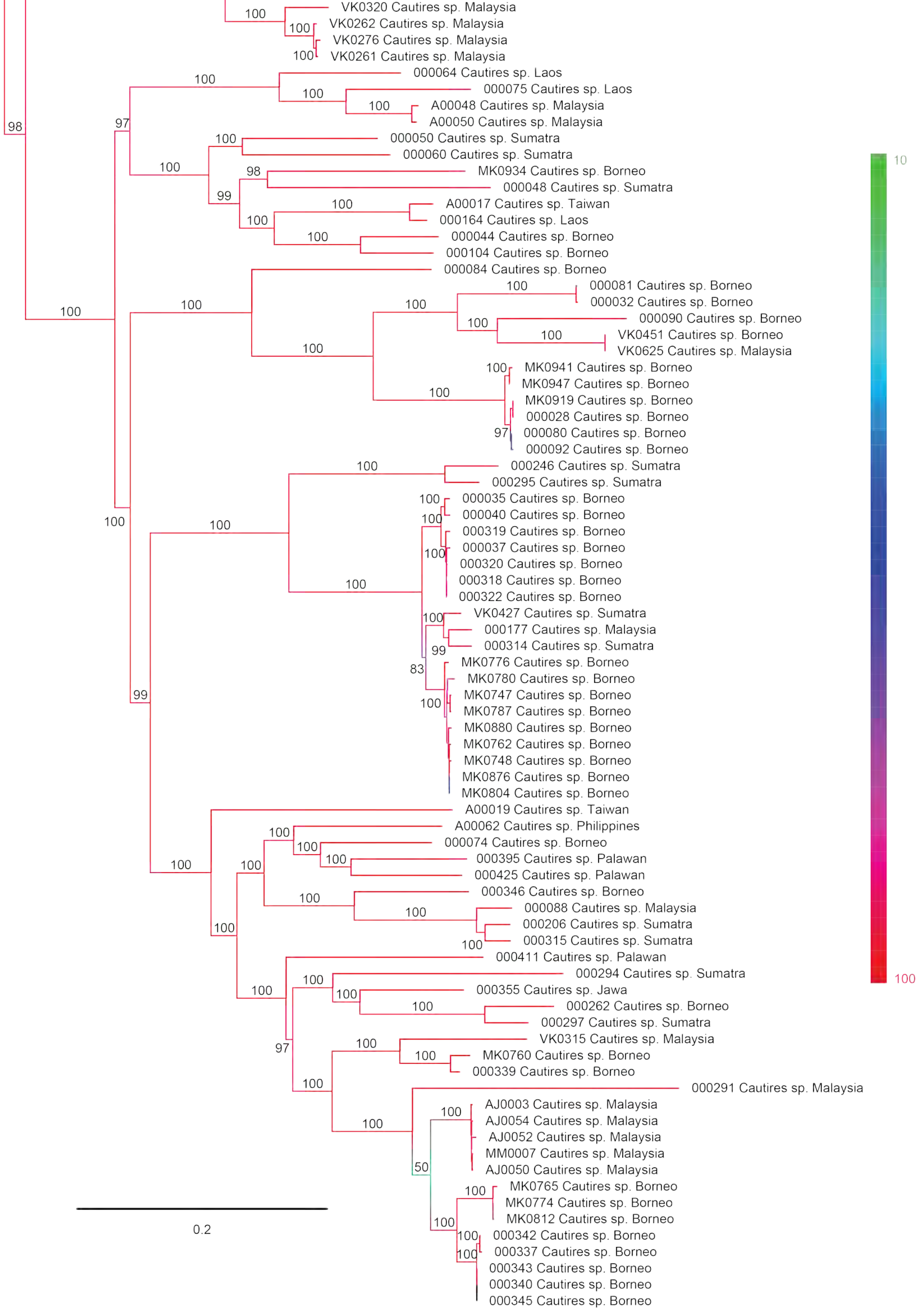

Figure S1. Maximum likelihood tree recovered by the analysis of the Metriorrhynchini full dataset.

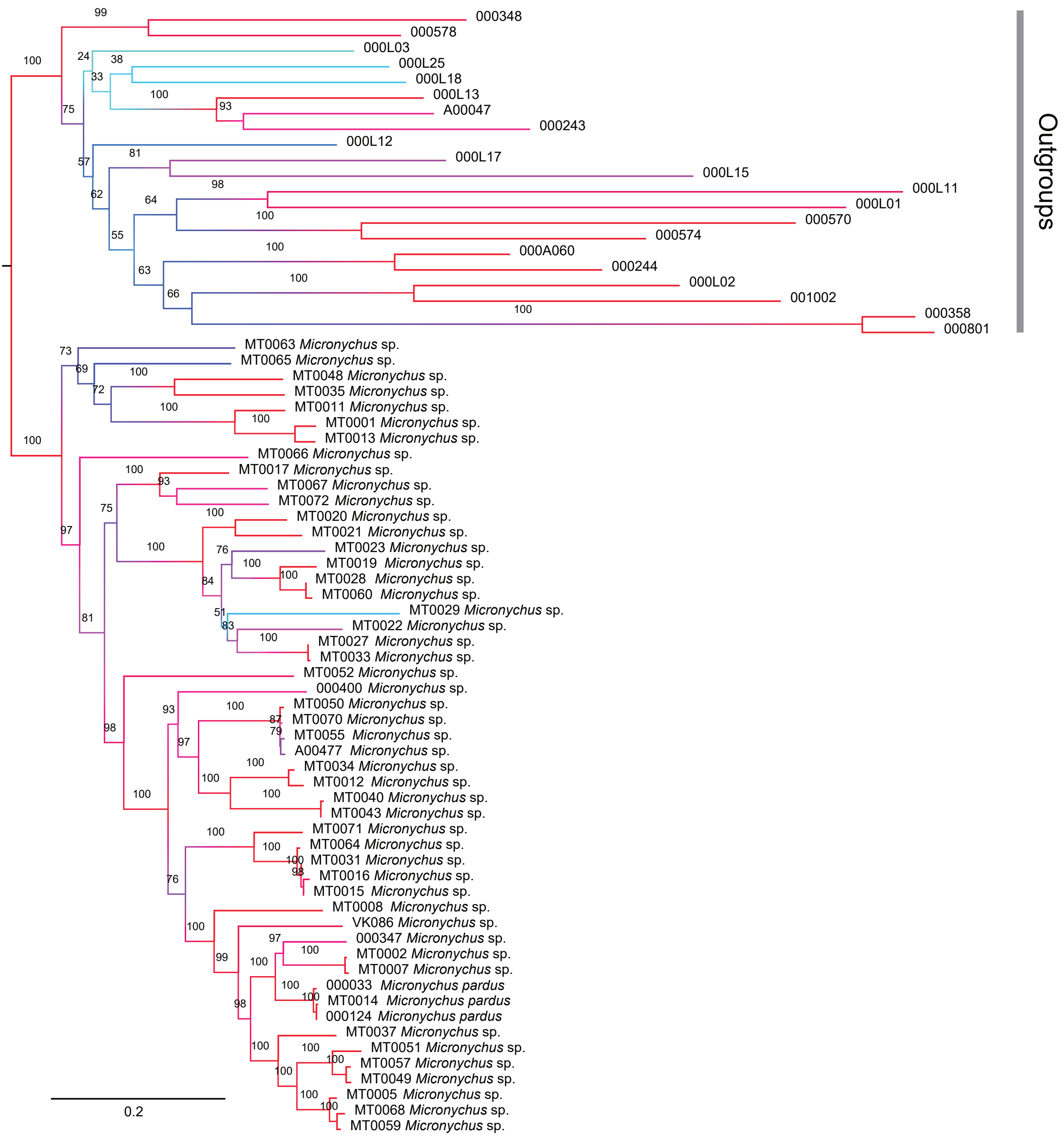

Figure S2. Maximum likelihood tree recovered by the analysis of the Calochromini full dataset.

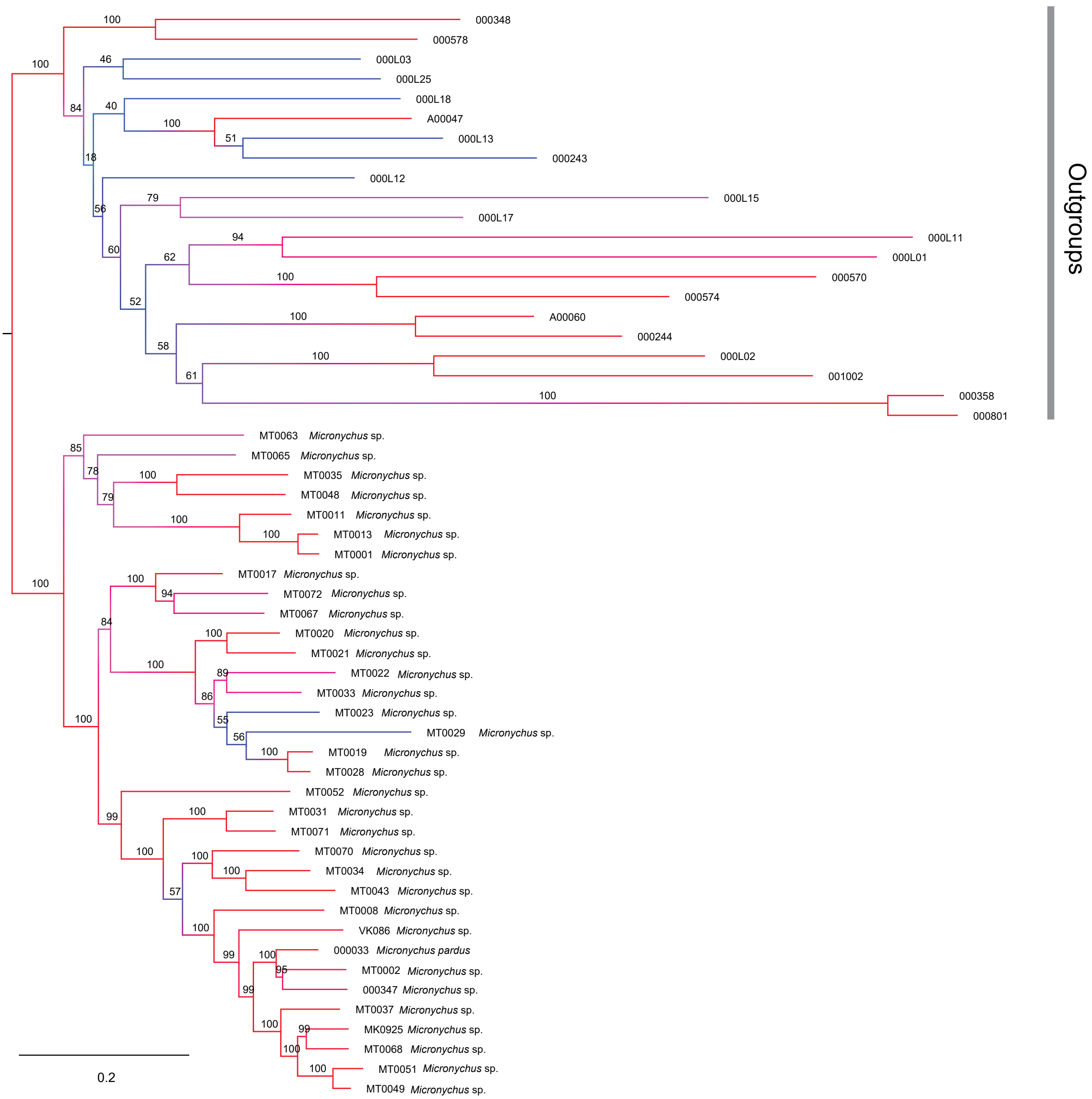

Figure S3. Maximum likelihood tree recovered by the analysis of the Calochromini reduced dataset.

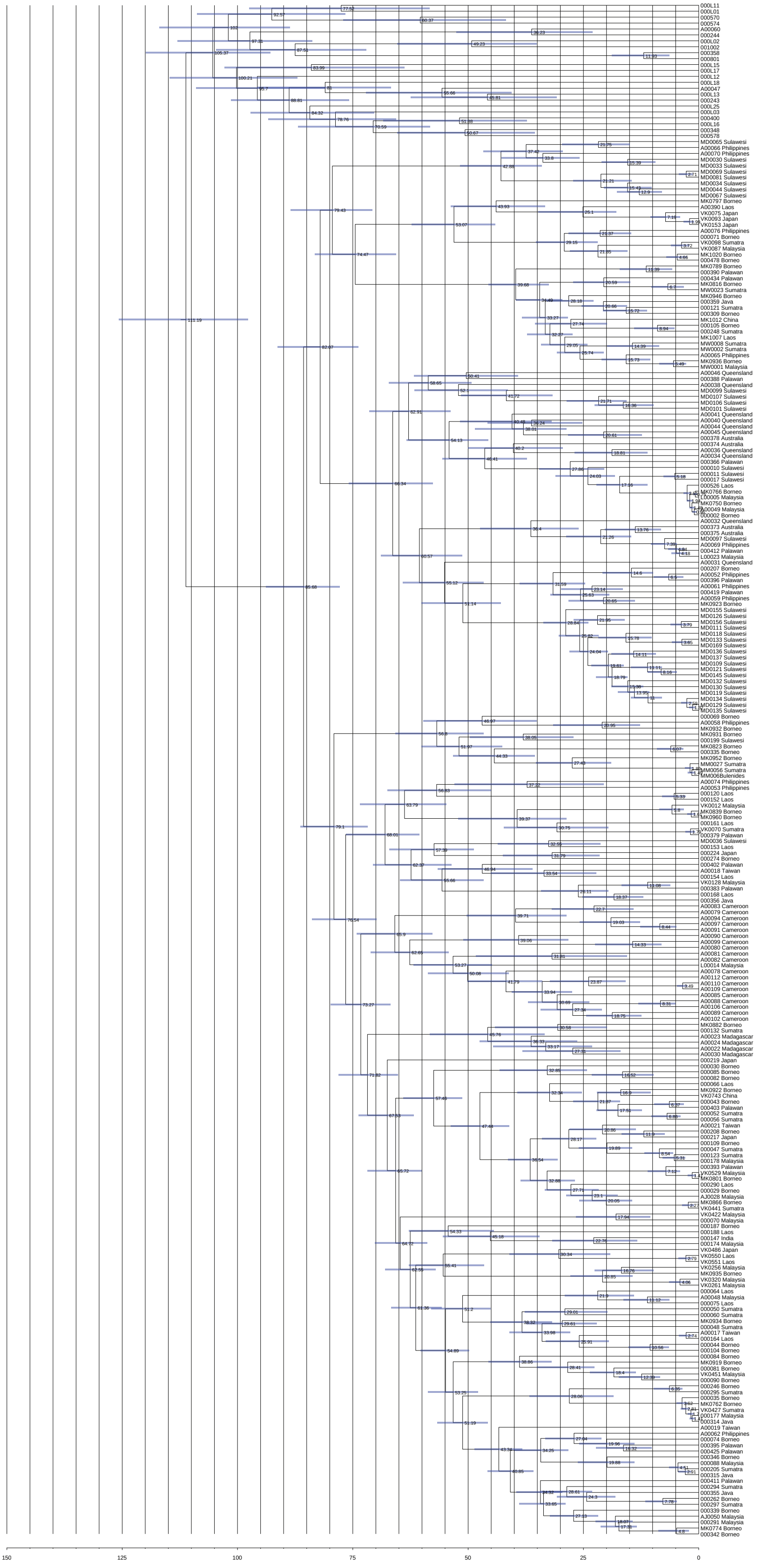

Figure S4. Time-calibrated, maximum clade credibility tree computed in BEAST from the reduced dataset with the topology constrained to the results of the maximum-likelihood analysis, the full resolution version of the Fig. 2A.

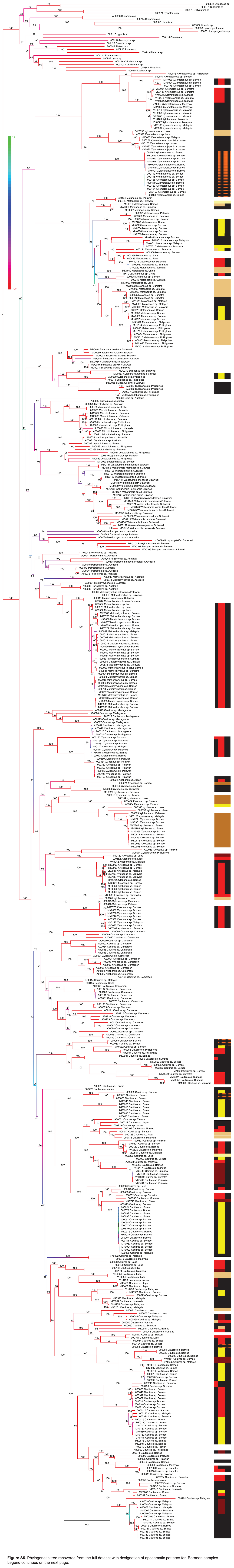

Figure S5. Phylogenetic tree recovered from the full dataset with designation of aposematic patterns for Bornean samples. Legend continues on the next page.

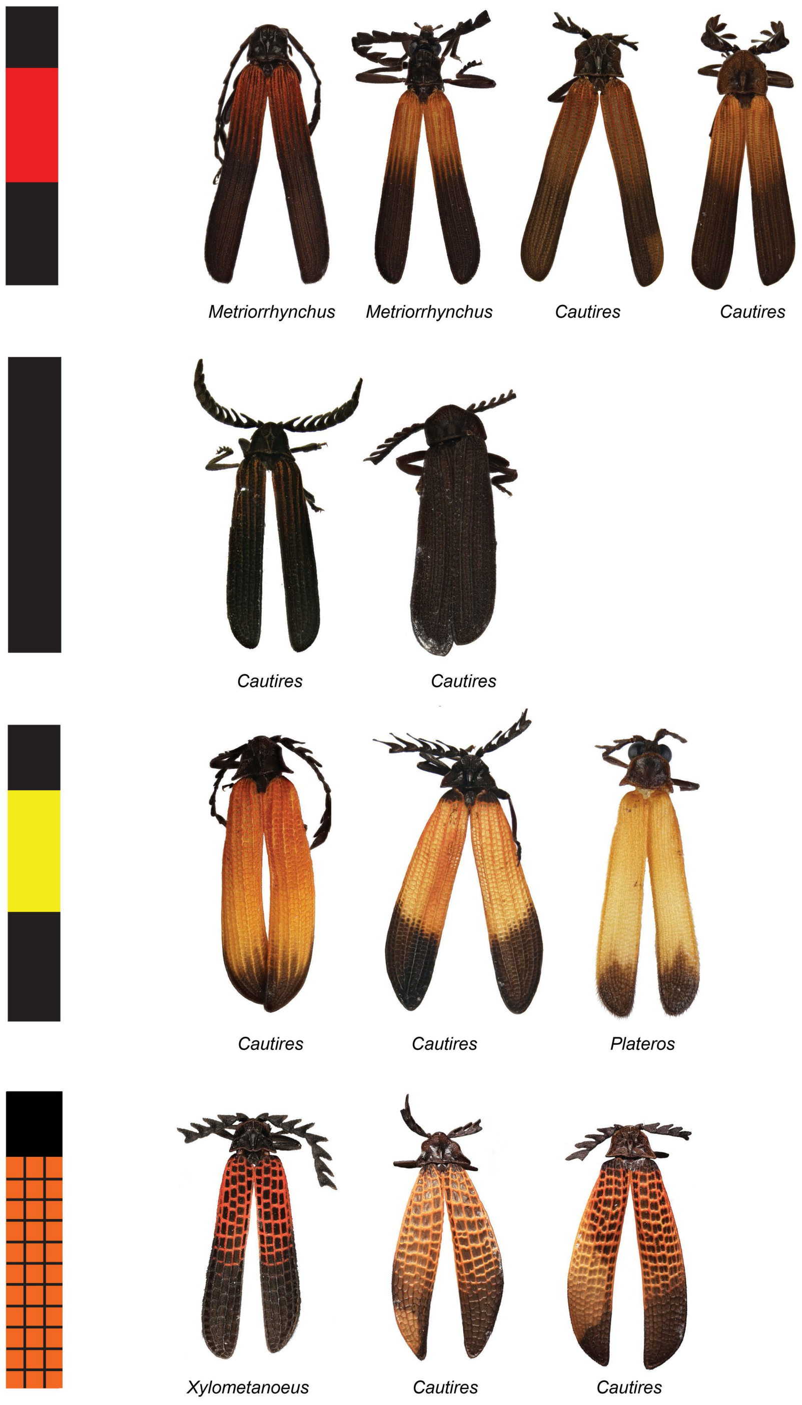

Figure S5. Phylogenetic tree recovered from the full dataset with designation of aposematic patterns for Bornean samples.

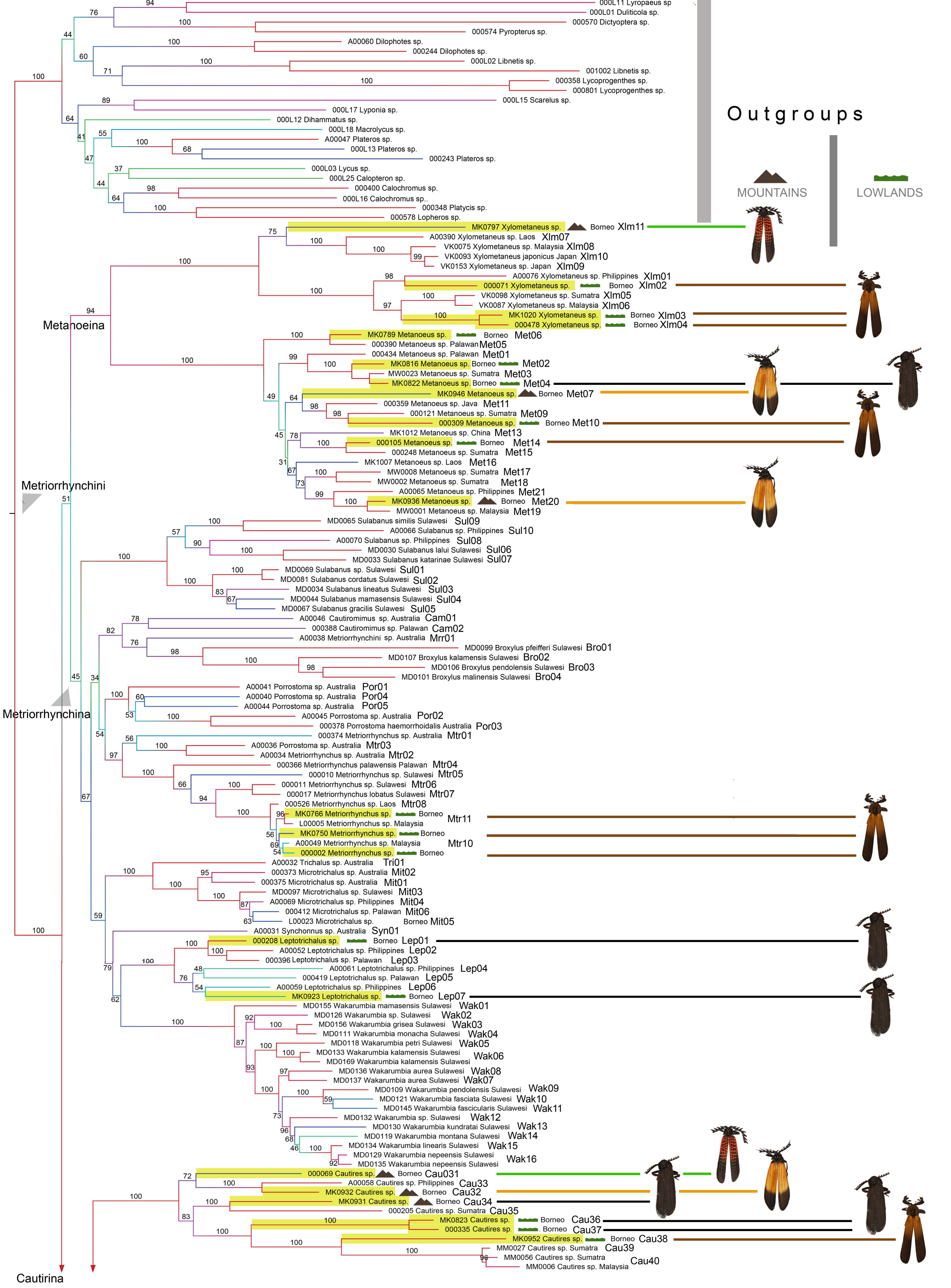

Figure S6. The distribution of the categorized aposematic patterns. Green, Yellow, Brown and Black lines represent species with the reticulate pattern, yellow/black pattern, dark red/brown coloration, and black coloration respectively. Brown pictograms represent samples from a higher elevation, green pictograms represent samples from lowlands. The figure continued on the next page.

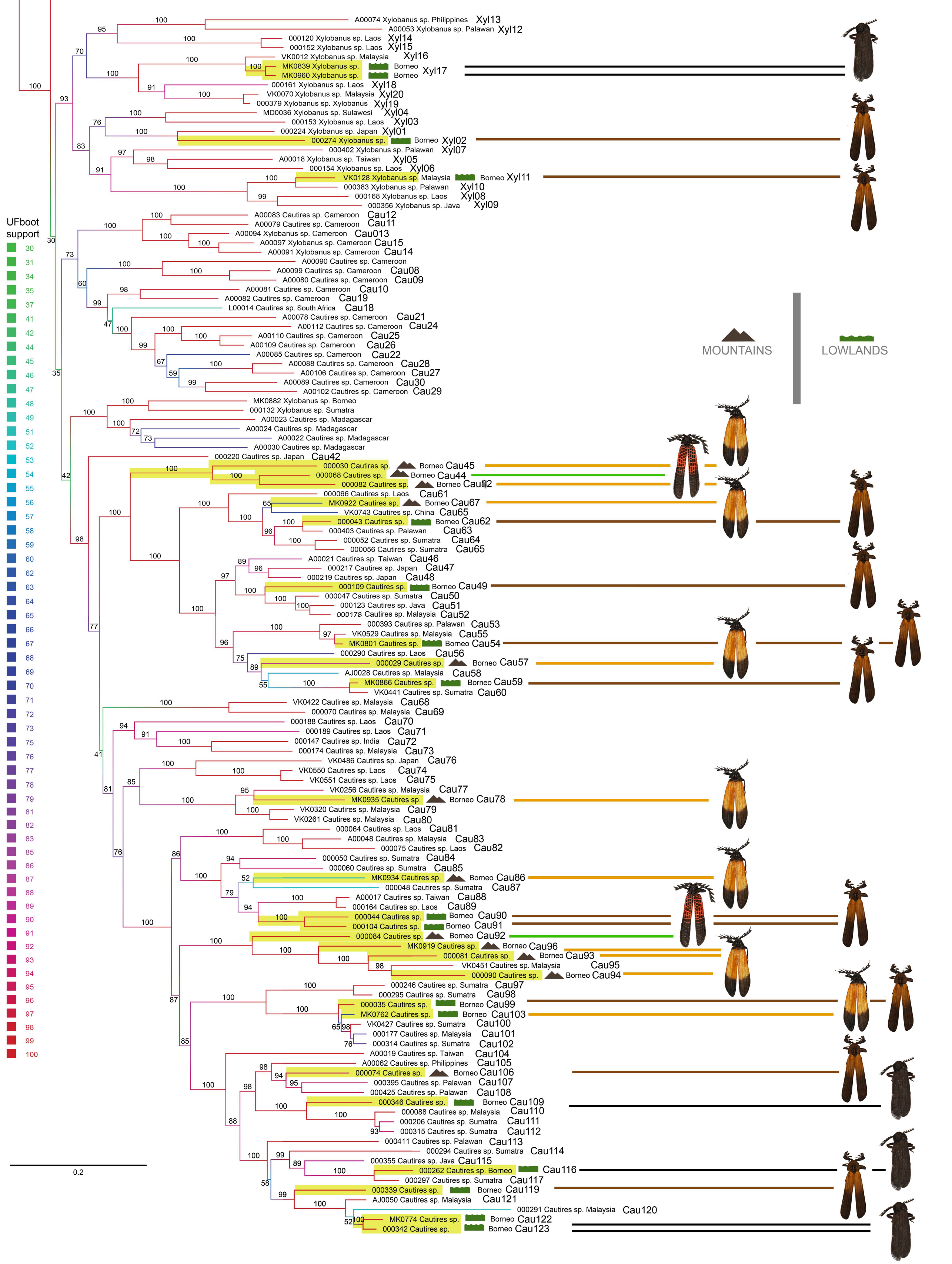

Figure S6. The distribution of the categorized aposematic patterns. Green, Yellow, Brown and Black lines represent species with the reticulate pattern, yellow/black pattern, dark red/brown coloration, and black coloration respectively. Brown pictograms represent samples from a higher elevation, green pictograms represent samples from lowlands.
